# Big Data and Artificial Intelligence in Cancer Drug Discovery: Promise, Challenges, and Emerging Opportunities

**DOI:** 10.3390/cancers18182939

**Published:** 2026-09-10

**Authors:** Fakhar U. Singhera, Justin M. Overhulse, Terrence M. Lee, Jonathan E. Katz, Jerry S. H. Lee, Charles E. McKenna

**Affiliations:** 1 Mork Family Department of Chemical Engineering and Materials Science, University of Southern California, Los Angeles, CA 90089, USA; fsingher@usc.edu; 2 Ellison Medical Institute, Los Angeles, CA 90064, USA; katzj@usc.edu; 3 Department of Chemistry, University of Southern California, Los Angeles, CA 90089, USA; joverhul@alumni.usc.edu (J.M.O.); tmlee@usc.edu (T.M.L.); 4 Department of Pharmacology and Pharmaceutical Sciences, Alfred E. Mann School of Pharmacy and Pharmaceutical Sciences, University of Southern California, Los Angeles, CA 90033, USA; 5 Department of Medicine, Keck School of Medicine, University of Southern California, Los Angeles, CA 90033, USA; 6 Department of Quantitative and Computational Biology, University of Southern California, Los Angeles, CA 90089, USA

**Keywords:** cancer, big data, drug discovery, machine learning, artificial intelligence, high throughput screening, high content screening, virtual ligand screening, clinical trials, real world data, real world evidence

## Abstract

Oncologic drug development is lengthy (~14 years) and expensive (~1.2 billion USD) with low clinical trial success rates (4.1%). Big data and artificial intelligence (AI) are widely proposed as tools to address these challenges. In this review, we examine the current performance and future potential of big data and AI applied to preclinical discovery and development, clinical trials, and the regulatory approval process. We first examine the data foundation required for effective AI, including data harmonization, data commons, and analytical tools. We then assess preclinical applications spanning target identification, compound-library curation, virtual ligand screening, generative chemical design, and high-throughput and high-content screening. In clinical development, we consider the use of big data and AI for outcome prediction, trial design, external and synthetic control arms, adaptive monitoring, and in silico trials. Finally, we discuss how post-approval electronic health records can generate real-world data and real-world evidence to support drug repurposing and improve future oncology drug discovery. Big data is conventionally characterized by a series of “Vs.” In this review, we have used seven “Vs” spanning descriptive and constraining properties of big data and a singular outcome. We have proposed an eighth, Vernacular, a constraint defined as the combined alignment of data semantics and terminology, data representation, data exchange, and data governance across heterogeneous, independently generated datasets to promote interoperability and combined analysis. Although cancer data exhibit substantial Volume, Velocity, and Variety, they remain distributed across fragmented repositories that often cannot be readily integrated. We conclude with a discussion of tabulated resources currently available for the application of big data and AI to oncologic therapeutics.

## Simple Summary

Only 4.1% of potential cancer therapeutics reach the clinic despite taking roughly fourteen years to develop at a cost of more than a billion USD. Large datasets and artificial intelligence (AI) are promising new tools to improve the odds. Cancer drug discovery produces enormous amounts of data related to cancer targets, candidate drugs, and patients. Separate datasets often use different terms for the same concept, so combining them proves harder than collecting them. Big data is described by seven “V” terms used as descriptors, constraints, and outcomes, and we review how researchers apply AI across cancer drug discovery. Here we add another, Vernacular, a property describing how well separate datasets share the principles, terms, data formats, and exchange methods they need to be analyzed together. We close this review with a list of resources to aggregate data and apply AI to cancer drug discovery.

## 1. Introduction

Cancer is one of the most prevalent diseases globally with, as of 2022, 20 million new cases yearly with approximately 9.7 million deaths annually [1]. In the United States (US) alone, over 2.1 million new cases are recorded every year, leading to approximately 625,000 deaths annually [2]. According to the National Cancer Institute (NCI), 39.2% of individuals will be diagnosed with some type of cancer during their lifetime, with a pan-cancer 5-year survival rate of 70.5% based on 2021–2023 data [2]. This figure, however, obscures the profound disparities between cancer types, especially those with low survival rates such as pancreatic, lung, and esophageal cancers [3]. Despite 51.4 billion US dollars (USD) in global public and philanthropic cancer research funding between 2016 and 2023 [4], the Food and Drug Administration (FDA) approved only 11 to 19 novel oncology molecular drugs and biologics per year between 2018 and 2023 as reported by the Oncology Center of Excellence, with 2025 staying within this range at 16 [5,6,7,8,9,10]. Much of this funding supports preclinical research, leaving the burden of official approval to be financed by the private sector [4].

Cancer is a clinically diverse disease with fundamental biological complexity reflected in both intertumor and intratumor heterogeneities linked to genomic, epigenomic, environmental, and biochemical etiologies [11]. This heterogeneity poses significant challenges to oncological drug discovery and development, contributing to lengthy timelines, high costs, and high failure rates. Regulatory requirements to ensure safe and effective drugs add to the data burden with the 1938 Food, Drug and Cosmetic Act requiring premarket evidence of safety and the 1962 Kefauver–Harris amendment introducing requirements for well-controlled clinical trials and adverse event reporting for new drug approvals [12]. The safety and efficacy requirements of these regulations lengthened the time needed for the drug approval because of the extensive data required to be submitted to the FDA.

Published estimates of drug development costs vary approximately ninefold according to a review conducted by Morgan et al. in 2011 [13]. For oncology, Sertkaya et al. estimated an average cost of 85 million USD per approved drug, rising to 595 million USD when failed programs are included and rising to 1.2 billion USD with the addition of capital costs. Development from target identification all the way to FDA approval takes 171 months or 14 years on average [14]. The sevenfold difference between approved drug costs (cost alone vs. failed programs included) is reflected by the 4.1% success rate of oncological drugs getting approved [14,15,16]. Based on ClinSR, neoplasm development programs were tracked from 2000 onward and studied for the probability of success from one phase to the next. The most recent window (2017 to 2025) shows failure rates of 64.7%, 79.2%, and 43.7% for success of phase I, II, and III, respectively, with concomitant cost increases at each trial phase [14,16].

Improved data-based prediction is essential to reduce this failure rate and decrease time with no negative impact on safety. This challenge requires harnessing the full potential of big data and artificial intelligence (AI) tools through the full pipeline. However, the binding constraint is not the amount of available data or the timeframe to generate new data but rather ensuring the accuracy, value, and interoperability of the data. Recent reviews cover the entire drug discovery pipeline, with each covering different sections like target identification and early clinical development [17], preclinical discovery through translation [18], and the entire pipeline including post-marketing surveillance based around the Design-Make-Test-Analyze (DMTA) cycle [19,20].

### Structure of This Review

This review focuses primarily on small molecule therapeutics across the oncology drug discovery pipeline, with biologics mentioned briefly in Section 4.7. Biologics are of major clinical importance in oncology, but the data commons, compound libraries, and computational tools described in this review are largely specific to small molecules with their biological counterparts undergoing definition in real time. This review provides readers with resources to educate and familiarize themselves with the process to better utilize the tools and databases mentioned.

**Figure 1:**
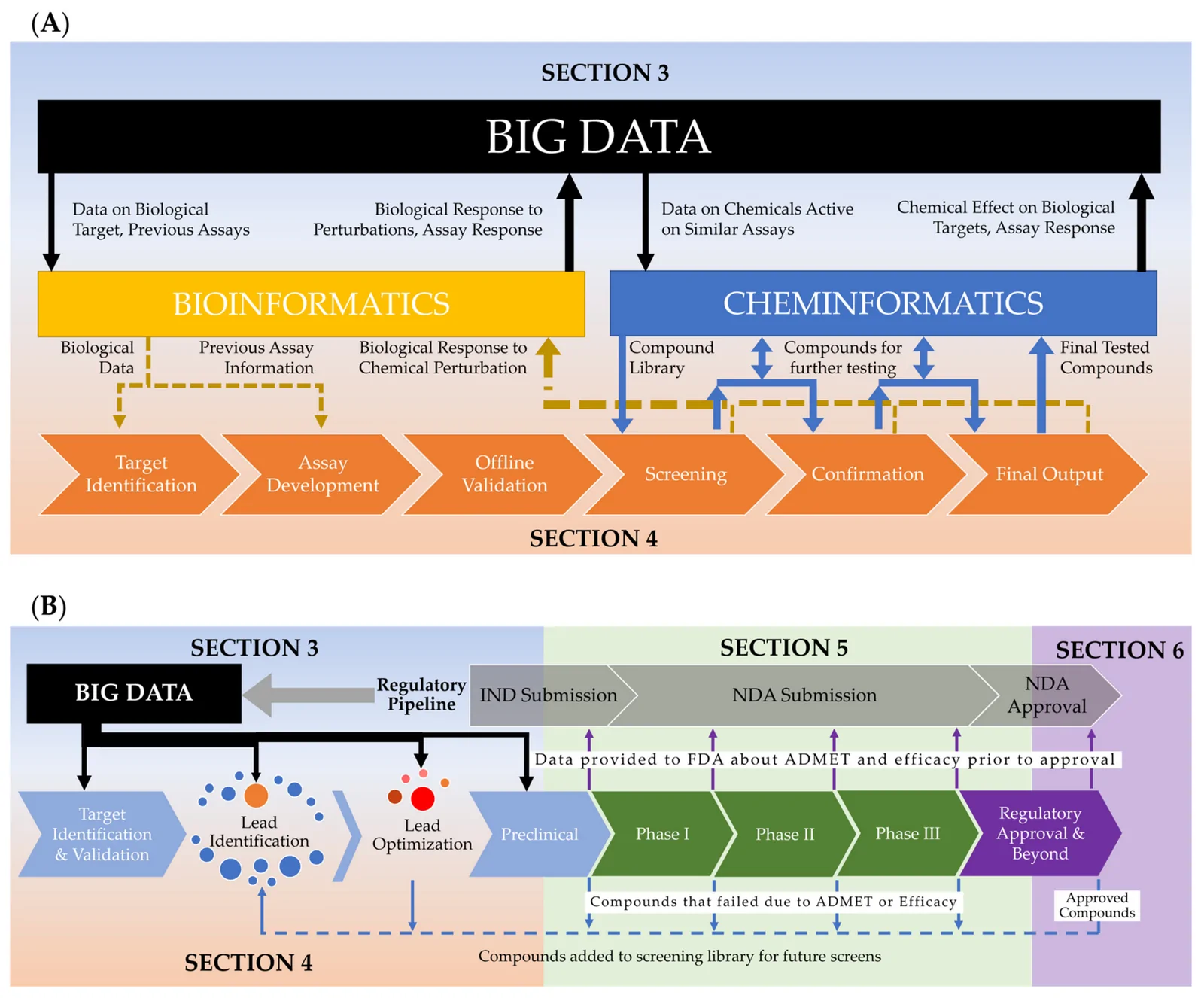
The role of big data in the drug discovery pipeline mapped to the corresponding sections of this review: (**A**) Preclinical cancer drug discovery. This covers the information in Section 3: Data collection and harmonization, available data commons for cancer drug discovery covering both bioinformatics and cheminformatics, and in Section 4: Target identification, assay development for both in vitro and in silico compound testing. The figure emphasizes the feedback loops integrated into this process and emphasizes the movement of information from data sources to inform the drug development and the lessons learned for future drug programs. (**B**) The entire drug discovery pipeline highlighting the transition from preclinical cancer drug discovery (Section 3 and Section 4) towards clinical trials (Section 5) and regulatory approval and beyond (Section 6). This figure highlights the interaction between the different stages of drug discovery along with the continuous information provided to the FDA regulatory pipeline and the iterative role of the data in the drug approval process and the feedback between the different stages.

The review follows the pipeline shown in Figure 1. Section 2 defines the terms and evaluation standards used throughout the review. Section 3 covers the data infrastructure underlying the entire drug discovery pipeline: harmonization standards, governance, cancer data commons, and visualization and analysis tools. Section 4 focuses on medicinal chemistry and preclinical drug discovery (Figure 1), covering the following: target identification (Section 4.1), compound management (Section 4.2), virtual ligand screening (Section 4.3), iterative library curation and in silico screening (Section 4.4), chemical synthesis using generative design (Section 4.5), assay development through high throughput and high-content screening (Section 4.6), and biologics (Section 4.7). Section 5 addresses clinical trial development, covering the following: trial outcome prediction (Section 5.1), trial design (Section 5.2), external and synthetic control arms (Section 5.3), adaptive monitoring (Section 5.4), and in silico trials and digital twins (Section 5.5). Section 6 examines the role of electronic health records (EHR), real-world data (RWD), and real-world evidence (RWE) after therapeutic approval and market entry. Section 7 identifies the computational skills that big data and AI require and points to learning resources to build familiarity with the entire pipeline.

## 2. Terms and Evaluation Standards

Section 2 defines the terminology, analytical framework, and evaluation criteria used throughout this review.

### 2.1. Defining Big Data

Big data refers to datasets too large and complex for conventional processing, requiring advanced computational analysis to reveal heuristically valuable patterns, trends, and associations [21]. The pharmaceutical industry has adopted the definition of big data that Doug Laney put forth in 2001, consisting of “the 3 Vs”—Volume (amount), Velocity (rate of data generation), and Variety (types of data) that describe the data [22]. Subsequent expansions add the terms that constrain the data: Veracity (accuracy and trustworthiness), Variability (differences within and between sources), and Visualization (display and interpretability), together with the outcome term, Value (usefulness for the specific problem), resulting in a total of “7 Vs” [23,24]. Later compilations also suggest many additional “V” terms, such as Vocabulary, covering the intra-dataset metadata defined by the data originator, covering structure, syntax, content, and origin, including schema, semantics, ontologies, and taxonomies [25].

Instead of Vocabulary, we propose a different eighth term, Vernacular, as an additional constraining property. We define Vernacular as a property of a data ecosystem: the extent to which independently generated datasets align sufficiently for joint analysis without curating each dataset separately. Vernacular resolves into semantic alignment (variable and conceptual consistency), representational alignment (compatible schemas, units and identifiers), exchange alignment (transmission between systems without bespoke conversion), and governance alignment (permission and consent requirements). Datasets do not need to align equally across the four dimensions, and high alignment in one does not mean that the others are well aligned. Therefore, Vernacular describes a degree of alignment rather than a binary determination of interoperability. Vernacular does not replace controlled vocabularies, ontologies, common data models, or interoperability standards, which can increase alignment across the dimensions of Vernacular through different mechanisms (Section 3.1).

Table 1 groups these seven properties and the Vernacular property proposed by the authors into three classes: descriptive, constraining, and outcome (Value). A dataset of substantial Volume, Velocity, and Variety can provide limited Value with poor Veracity, high Variability, and inadequate Visualization. Due to the inherent heterogeneity and Variability in biology, insufficient alignment of any of the four Vernacular dimensions can prevent reliable interpretation and integration of diverse datasets, limiting the Value of individual datasets that cannot be readily combined. Data acquires Value when these properties are sufficient for a specific decision rather than from scale.

### 2.2. Defining Data Terms

Two informatics terms recur throughout this review. Cheminformatics is the collection and analysis of large quantities of chemical data, formulas, structures, properties, spectra, and potential biochemical or biological activities [26,27,28], while bioinformatics conceptualizes biology with the organization and analysis of macromolecules, drawing on structures and functional genomics/expression data with heterogeneous data integration for applications including drug design [29]. For the purposes of this review, we draw on a narrower definition of bioinformatics by defining it as the collection and manipulation of data from in vitro and in silico screens focused on a specific target’s biochemical or biological activities. In drug discovery, these fields increasingly overlap, as chemical activity is interpreted together with the molecular and biological characteristics of the target or experimental model, as shown in Figure 1.

In addition to the informatic terms, several computational terms also recur throughout this review. AI refers to a set of algorithms allowing computers to solve problems that resist human-encoded rules. Machine learning (ML), a subset of AI, uses data to learn patterns and relationships that can be applied to prediction or classification, while deep learning (DL), a subset of ML, uses multilayer neural networks to learn increasingly complex representations of the underlying data. Both approaches can be applied through supervised learning, in which known inputs are mapped to known outputs, or unsupervised learning, in which underlying patterns and relationships are identified without predefined output labels [30]. Reinforcement learning optimizes toward a defined reward through trial and error, while generative models sample new instances from a learned distribution [31]. Both are encountered in this review in generative chemistry, antibody design, and synthetic control arm construction.

Foundation models are trained at scale on large, diverse datasets and can be adapted to downstream tasks [32]. Large language models (LLMs) are a common type of foundation model trained on large text datasets to interpret and generate natural language. Language models have been adapted to drug discovery by training on biological sequences and chemical representations such as Simplified Molecular Input Line Entry System (SMILES) rather than on text [33]. Multimodal architectures accept several data types (imaging, genetics, sequences, free text, etc.) simultaneously and learns relationships across them [34]. These distinctions matter because increasing model capacity can exploit greater Volume and Variety but also increases dependence on the Variability and the Vernacular of the underlying data, since capability is drawn from combining sources [32,34]. Computational sophistication therefore does not remove the data constraints defined in Section 2.1, a limit that recurs wherever these models appear, such as in structure prediction, image-based screening, and trial outcome predictions.

### 2.3. Aims and the Interrelatedness of the Pipeline

This review has three aims. First, it applies the “V” framework across the entire oncology pipeline, from target identification through regulatory approval and real-world use, rather than treating preclinical and clinical portions of the pipeline separately. Second, it proposes Vernacular as an eighth “V” and uses it to explain why datasets that are individually well curated resist joint analysis. Third, it argues that data integration rather than data generation constrains oncologic drug discovery, a claim that each section of the review builds upon and that is revisited in the conclusion.

Reviews conventionally draw the drug discovery pipeline (Figure 1) as a sequence from preclinical work (target identification, lead identification, lead optimization, preclinical model testing) to clinical development (phase I, II, and III trials and regulatory approval). Functionally, it operates as an iterative data network. Each stage consumes the data produced by previous stages and generates data that can be used in future programs. Regardless of outcome, each stage provides data that can inform target selection, biomarker definitions, and dosing information for new programs. Five junctions recur through this pipeline: data commons feeding target selection (Section 3 and Section 4.1), computational prediction feeding experimental screening (Section 4.4, Section 4.5 and Section 4.6), preclinical results feeding trial design (Section 4 and Section 5.2), trial data feeding regulatory review (Section 5 and Section 6.2), and real-world use feeding new discovery programs (Section 6 feeding back to Section 3, Section 4 and Section 5).

This structure determines where constraints arise, and the junctions between stages rather than the stages themselves are where Vernacular becomes consequential. At each junction, the question is not only whether the available data are accurate and valuable within the context in which they were generated but whether downstream users operating with different tools and conventions can also access, interpret, and integrate them.

### 2.4. Validation and Evaluation Standards

The increasing use of computational models across the oncologic drug discovery pipeline makes validation, bias, and reproducibility determinants of data Veracity and Value. Reported performance metrics need not reflect performance of new experimental or clinical data, especially for AI models using ML and DL, which learn from the training datasets, including any statistical correlations, biases, and technical artifacts [35]. These failures arise at three points: in the data a model learns from, during training, and after deployment.

Several limitations arise from the underlying data. Selection bias occurs when the development dataset is not representative of the population or the conditions in which the model is applied. Confounding can create associations between model inputs and outcomes that do not reflect causal relationships [36]. Missing data introduces additional bias when the probability of a measurement being absent depends on experimental, patient, or outcome characteristics [35]. In experimental datasets, batch effects (based on environment, instruments, reagents, operators) can cause models to learn the batch effects as predictive features [37]. These problems are important in oncology due to the heterogeneity of cancers, experimental platforms, and small patient subgroups.

Limitations can also occur during model training. Overfitting occurs when the model incorporates training data too well and identifies noise rather than the real patterns. Data leakage occurs when the training dataset includes information that would not be available, such as training data or additional fields, during model development and therefore inflates reported performance [38].

Further limitations arise after deployment. Dataset shift occurs when the new dataset results differs from the data used in training, while model drift is the deterioration in performance of a model as assays, population dynamics, and standard of care evolve [39]. These failures develop over time rather than at a single point; they express ongoing Variability temporally and remain invisible to one-time evaluation and thus require longitudinal tracking.

Evaluation therefore must extend beyond a single performance metric. Calibration determines whether predicted probabilities correspond to the observed outcomes, while interpretability identifies contributing features without establishing whether a prediction is valid [40,41]. Explainable AI (XAI) methods comprise interpretable models and post hoc attribution for opaque models, such as by assigning each feature an importance Value for a particular prediction. Interpretable models are preferred for high-stakes decisions since attribution explanations do not necessarily reflect the model they describe [40]. Regulators increasingly ask sponsors to state the basis on which a model informed a decision. Cross-validation estimates performance within similar data, while external validation on independently collected datasets provides stronger evidence of generalizability, and prospective monitoring becomes necessary once models operate in changing environments [42]. Reporting standards such as TRIPOD + AI make these properties assessable and improve transparency of model development and evaluation [43,44].

This review distinguishes four levels of validation evidence. Computational proof of concept establishes performance on retrospective data in silico. Experimental validation confirms in silico predictions in in vitro or in vivo models. Prospective validation forecasts outcomes before they occur and are used in experimental or clinical settings. Regulatory validation requires pre-specifications against defined contexts of use. Each level constrains what a reported performance figure can support, and this review notes the level reached wherever the underlying evidence allows (Table 2). The table relates the AI approaches discussed in Section 2.2 with the validation standards in this section and applies them to the material discussed through this review. Because most of these limitations trace how independently generated datasets are collected and combined, Section 3 turns to the infrastructure governing them.

## 3. Data Infrastructure for Cancer Drug Discovery

Effective drug discovery depends on the ability to collect, organize, and interpret large Volumes and Varieties of data while maintaining their Veracity, accessibility, and interoperability across different data types and stages of development. Cancer research is voluminous and heterogeneous, incorporating data from genomics, proteomics, clinical outcomes, and pharmacological activity [45,46,47]. The translational utility of these data is dependent on the ability of researchers to effectively utilize them. As a result, international organizations and communities have developed frameworks to provide standardized data structures, terminologies, exchange mechanisms, and data deposition guidelines [48,49,50,51]. These standardization principles underpin the data repositories and various data commons that support drug discovery and increase shared Vernacular across otherwise heterogeneous sources.

### 3.1. Integration, Harmonization, and Sharing of Data Sources for Cancer Drug Discovery

Drug discovery and oncologic drug utilization consistently generate large Volumes of data at high Velocity, and these data can be difficult to store, integrate, and analyze across independently generated sources.

Cancer staging provides an example of this limitation. Large datasets examining cancer stage at diagnosis are valuable for determining treatment, survival, interventional efficacy, and regional disparities. However, there are several standards, including tumor-node-metastasis systems (TNM), SEER Summary Stage (SSS), Essential TNM, and Condensed TNM, which are not easily interchangeable [52,53]. In particular, SSS, a mandated component of US central registries, permits the recording of missing information as negative, risking underreporting compared to TNM, which requires comprehensive detail and is consequently used less widely [52]. This risk of underreported stage determination for registries using SSS compared to TNM compromises larger regional and country epidemiological studies, highlighting the need for harmonizable standards that preserve the meaning of the underlying variables when datasets are combined. Thus, a common label such as “stage” does not ensure shared meaning or representation. Increasing Vernacular would require that differences in definitions and missing-data conventions are explicitly mapped rather than silently reconciled. The failure here is semantic and representational rather than one of data transmission, as the datasets describe the same clinical concept, but the definitions and conventions used to encode it differ. Combining them without preserving these differences can increase Volume while decreasing Veracity and consequently Value.

Existing interoperability frameworks address different components of this property. The Findable, Accessible, Interoperable, and Reusable (FAIR) principles establish broad goals for scientific data to improve reusability by both humans and computational systems but do not prescribe a single terminology, data model, or exchange mechanism [51]. FAIR establishes principles for each resource independently, while the proposed Vernacular property describes the degree of alignment between two independent resources and their ability to be used together. Two datasets can independently satisfy FAIR but still resist joint analysis due to a lack of shared vocabulary between the datasets. The controlled vocabularies and ontologies supply semantic alignment by standardizing concepts and their relationships, without prescribing how those concepts are represented or exchanged.

Common data models and research standards supply representational alignment. The Observational Medical Outcomes Partnership (OMOP) Common Data Model transforms heterogeneous observational healthcare data into a standard structure and vocabulary [50]. Across six databases, Voss et al. mapped 96–99% of condition records and 90–99% of drug records to standardized concepts, though mapping does not correct errors or biases already present in the data [50]. OMOP raises representational alignment without considering Veracity, constraining pooling. The Clinical Data Interchange Standards Consortium (CDISC) standardizes the collection, organization, analysis, and regulatory submission of clinical and nonclinical data through a series of interrelated standards [48]. Both OMOP and CDISC increase the shared Vernacular within different portions of healthcare and drug development, but neither spans the entire drug-discovery pipeline.

The Health Level Seven Fast Healthcare Interoperability Resources (FHIR) supplies exchange alignment by standardizing the electronic exchange of healthcare information [49]. This exchange does not guarantee equivalent representation or interpretation. Explicit harmonization between OMOP and FHIR produced only moderate agreement at the model level (Fleiss’s inter-rater agreement of κ = 0.50) and fair agreement at the property level (κ = 0.21) [54]. Two established standards can exchange data while still requiring additional mapping for combined use, illustrating that interoperability does not ensure alignment for joint analysis.

Data harmonization has been advanced by the creation of common regulatory standards, such as the International Council for Harmonisation’s (ICH) Common Technical Document (CTD) [55]. The CTD standardizes the organization of quality, safety, and efficacy information for regulatory authorities across the three major pharmaceutical regulatory regions, the European Union (EU), the US, and Japan, reducing the need to reformat the same information for different agencies [55]. This dossier is maintained by the ICH of Technical Requirements for Pharmaceuticals for Human Use and has been periodically updated since its implementation in 2002; it has greatly expedited the drug submission and review process [55]. Lee et al. discuss the importance of large amounts of data collected and reported uniformly to allow for ease of analysis [56].

Taken together, FAIR, controlled terminologies, OMOP, CDISC, FHIR, and the CTD illustrate how independently developed standards can increase different components of shared Vernacular, while no single framework spans the entire oncologic drug discovery pipeline. Vernacular therefore describes the collective degree of alignment achieved across these components rather than an additional standard that must itself be adopted.

### 3.2. Governance, Consent, and Permitted Use in Oncological Drug Discovery

The Velocity and Volume granted by using big data fo the storage, processing, and analysis of cancer biomedical research data make governance alignment, the fourth Vernacular dimension, a determinant of which datasets can be combined. Two datasets can be semantically, representationally, and technically compatible yet be unable to be used for joint analysis due to consent, access, or permitted uses. Data governance therefore determines the storage, exchange, access, consent, and permitted use of patient data.

The Health Insurance Portability and Accountability Act (HIPAA) in the US and General Data Protection Regulation (GDPR) in the EU illustrate governance constraints across different jurisdictions. HIPAA regulates protected health information (PHI) held by covered entities and businesses and regulates research use under defined conditions only [57,58]. Unlike GDPR, HIPAA does not impose requirements on geographic requirements on data storage if other conditions are met. GDPR designates health and genetic information as special-category data and imposes additional requirements for transfer outside of the EU [59,60]. This shows the legal constraints of governance on what could otherwise be technically interoperable cancer datasets.

US government databases like VPODC, and particularly NIH-funded databases and tools like TCGA, TCIA, and GDC, each have data intake procedures involving de-identification procedures that comply with HIPAA or the NIH Genomic Data Sharing Policy [61]. TCGA and GDC feature layers of access, with both featuring open access datasets primarily composed of high-level, non-individual, or summarized and aggregated data as well as controlled access that grants access towards low-level data like whole, raw, and individual datasets that have a higher risk of re-identification [62,63]. These access tiers are a governance boundary because two datasets accessed under different tiers cannot be pooled by a single investigator without permission allowing equal access.

While these systems comply with government standards of patient protection, studies have successfully re-identified patients at the controlled-access levels from raw genomic data at controlled access levels and even from open-access levels from aggregated statistics [64]. Homer et al. demonstrated in 2008 that aggregate allele frequencies alone could reveal whether a specific individual belonged to a study cohort, and NIH responded by moving summary-level data, including *p*-values and genotype counts, from open to controlled access on dbGaP [65]. Consequently, numerous research arms focus on developing, implementing, and measuring the efficacy of security procedures such as encryption and differential privacy to improve privacy protection in the current age of big data genomics in the drug discovery process [64].

Section 2.1 notes that governance alignment is institutional and legal, and thus no standard raises it. Investigators have turned toward federated approaches to comply with governance constraints and train models without moving underlying data in both public consortia and private databases (Section 3.3). Effective integration requires alignment in governance conditions alongside terminology, representation, and exchange.

### 3.3. Databases and Data Commons for Cancer Drug Discovery

Integration and harmonization of data sources are vital to increasing shared Vernacular across cancer drug-discovery resources. The main database for in vitro drug discovery in the US is PubChem, initiated with screening centers established by the National Institutes of Health (NIH) Roadmap Molecular Library Probe Centers Network (MLPCN) that harmonized data deposition [66]. Table 3 lists data commons developed by the NIH in the US and the European Molecular Biology Laboratory (EMBL)—European Bioinformatics Institute (EBI) in Europe to aid in both cancer and general target identification and drug discovery. Table 3 presents these as established repositories spanning chemical, structural, genomic, proteomic, pharmacological, and clinical data relevant to multiple stages of drug discovery and reports for each entries, access tier, update status, funder, and the purpose. All figures were verified against each resource on the date given in the table footnote.

Cancer research has benefited from data commons by integrating data and observations across varying disciplines. The NCI and the National Human Genome Research Institute came together from 2004 to 2014 to spearhead The Cancer Genome Atlas (TCGA), which contains genomic data for 33 cancer types and has been used by researchers to identify unique and pan-cancer targets based on genomic, epigenomic, transcriptomic, and proteomic alterations [67,68]. An analysis of 9125 TCGA tumors found that 89% contained at least one driver alteration in ten canonical signaling pathways, 57% contained at least one alteration potentially targetable by an available drug, and 30% contained multiple potentially targetable alterations [69]. These findings demonstrate how increased Volume and Variety can reveal therapeutic opportunities across cancers. TCGA was eventually combined with the Therapeutically Applicable Research to Generate Effective Treatments (TARGET) and other cancer datasets to form the Genomic Data Commons (GDC) in 2016, representing over 50,000 cases [70]. These large datasets support investigations that no single study could power, for example by investigating the expression levels of different proteins in varying cancers such as in acute myeloid leukemia. One analysis of treatment-naïve myeloid leukemia patients showed that overexpression of PDK2/3 was a poor prognostic factor [71].

The utility of these data commons extends beyond repeating previous analysis. Combining sufficiently harmonized datasets can increase the Volume and Variety of observations available for identifying potential candidate targets against a single cancer or pan-cancer by using molecular associations and testing for associations beyond the originating dataset. Existing data commons can provide additional utility when combined into larger available datasets to add statistical power and biological heterogeneity, dependent on the compatibility and quality of the data. In 2014, the NCI created the Cancer Research Data Commons (CRDC), which combined basic science, preclinical, cloud computing, analytics, and data visualization to provide high-Volume, high-Variety data alongside tools for visualization and analysis [46]. Another database, the NCI’s Cancer Target Discovery and Development Network (CTD^2^), builds on genomic resources by adding functional therapeutic data and contributing to bridging target identification and preclinical drug discovery [72]. Increasing dataset size alone does not ensure better AI performance, and the constraint appears with the NCI’s own databases. Kim et al. report that NCI and NIH repositories operate under different data standards, harmonization methods, and metadata requirements which limits the ability to pool and compare all the data they hold [46]. Repositories built under common governance therefore still differ in annotation, terminology, experimental or clinical bias, and processing technique, and combining datasets without accounting for these differences can introduce the selection bias and batch effects described in Section 2.4.

The commons described above depend on open deposition. However, much of the data generated in oncologic drug discovery is proprietary and never deposited into any publicly accessible database. Public-private consortia such as Accelerating Therapeutics for Opportunities in Medicine (ATOM) pool otherwise separate databases from government, industry, and academia into a shared preclinical modeling resource [73]. If this pooling is not possible, then institutions can form federated learning models without sharing the underlying data. The MELLODDY project, consisting of ten pharmaceutical companies (including AstraZeneca, Bayer, GSK, and others), trains a combined model using more than 2.6 billion confidential activity measurements covering over 21 million compounds and 40,000 assays [74]. All ten partners saw modest improvements in their models. Each partner’s baseline models on siloed data already achieved areas under the precision-recall curve of 0.6–0.8 with R^2^ values of 0.3–0.4. The federated model improved the Relative Improvement of Proximity to Perfection by 4% and 2%, respectively. The coverage of conformal efficiency increased by a median of 12% for all the partners [74]. Federated learning can therefore improve access to otherwise siloed datasets, but it does not eliminate differences in terminology, data representation, assay design, or data distributions between participating institutions. Federated learning carries its own costs, with repeated model exchange between institutions, increasing communication and coordination overhead. Model gradient sharing alone does not eliminate the risk of data reidentification [75], and the models can still drift with population level changes in the datasets. In the framework proposed here, it is a governance workaround rather than an alignment mechanism, as it removes the requirement to move data across institutional boundaries.

**Table 3:** Selection of common databases and data commons for cancer drug discovery.

Database/ Data Commons	Entries ^1^ (Year of Source)	Data Modalities	Update Status/ Access ^2^	Translational Utility
PubChem [76]	322M substances 119M compounds 295M bioactivities >1.2M assays (2025)	Chemical structures, bioassays, bioactivity	Continuous Open Access API and bulk download	Supports compound identification, bioactivity analysis, library curation, and preclinical drug discovery
ChEMBL [77,78]	2.9M compounds >18,600 targets >2.0M assays (ChEMBL_37: 2026)	Chemical structures, targets, bioassays, bioactivity	Periodic Open Access API and bulk download	Supports target prioritization, structure activity relationships (SAR), bioactivity prediction, and virtual screening
Protein Data Bank (PDB) [79]	>259,000 protein structures (August 2026 from website)	3D macromolecular structures	Weekly Open Access API and bulk download	Supports target characterization, structure-based design, docking, and virtual screening
The Cancer Genome Atlas (TCGA) [67]	~11,000 subjects; >20,000 samples (as of August 2026)	Multi-omic and clinical	Closed—see GDC Tiered ^3^ Access API and bulk download	Supports target discovery and biomarkers in pan-cancers and single cancers
Therapeutically Applicable Research to Generate Effective Treatments (TARGET) [80]	~6500 subjects; >10,000 samples (as of August 2026)	Pediatric multi-omic and clinical	Closed—see GDC Tiered ^3^ Access API and bulk download	Supports pediatric target discover and biomarkers in pan-cancers and single cancers
Clinical Proteomic Tumor Analysis Consortium (CPTAC) [45]	>1000 tumors; 10 cancer cohorts (as of August 2026)	Multi-omic and clinical	Ongoing Tiered ^3^ Access API and bulk download	Supports proteomics-driven target, pathway, and biomarker identification
Genomic Data Commons (GDC) [70,81]	>50,000 cases >22,000 genes >3M mutations 69 Primary Cancer sites 91 projects (August 2026 from website)	Multi-omic, biospecimen, molecular, and clinical	Continuous Tiered ^3^ Access API and bulk download	Supports cross-study integration, pan-cancer analysis, biomarkers, and computational resourcing
Cancer Research Data Commons (CRDC) [46,82]	>350 studies >134,000 subjects >2000 public tools and workflows (August 2026 from website)	Genomic, imaging, proteomic, bioassays, and clinical	Continuous Tiered ^3^ Access API and bulk download	Supports cancer data integration with basic science data, cloud computing, visualization, and analytics
Cancer Dependency Map (DEPMap) [83]	~1600 cell lines >2000 models 1150 genome-scale CRISPR >600 compounds (2025)	Multi-omic, drug response, cell models, and CRISPR	Quarterly Open Access Bulk downloads	Supports target dependency analysis, therapeutic vulnerabilities, and drug response prediction
Cancer Therapeutics Response Portal (CTRP) [47]	481 compounds; multiple cell lines (constant since 2016)	Drug response and cell line models	No updates Open Access Bulk downloads	Supports drug response modeling and mechanism of action analysis using known models and responses
Cancer Target Discovery and Development (CTD^2^) [72]	_ ^4^	Genomic, functional, and therapeutic	Ongoing Open Access Bulk downloads	Supports functional target validation and translation of genomic findings into therapeutic hypotheses
Veterans Precision Oncology Data Commons (VPODC) [84]	>110,000 subjects (2020)	Clinical, genomic, and imaging	Ongoing Controlled Access Bulk downloads	Supports precision-oncology research, clinical genomic associations, and real-world analysis

^1^ Abbreviations: M = million; PB = petabyte; TB = terabyte. ^2^ All these databases and data commons are AI ready based on API access or bulk downloads. This does not mean they are interoperable or can be jointly analyzed. ^3^ Tiered Access means that aggregate and summary data is available publicly, but individual data requires an application and permission. ^4^ No number provided, as CTD2 reports submissions of studies and could not be directly compared. All numbers verified 26 August 2026.

### 3.4. Analysis and Visualization Tools for Cancer Drug Discovery

The repositories described in Section 3.2 necessitate sophisticated analysis and visualization tools to extract meaningful insights. However, individual institutions/entities maintain these analysis and visualization tools, leaving the landscape fragmented. Overlapping functionalities, incompatible formats, and redundancies mean that researchers must build custom pipelines with multiple tools for data analysis instead of working within a single ecosystem. Efforts by the NCI in cancer discovery, including the CRDC, have started some implementation in this regard. However, these efforts remain incomplete because different datasets continue to require distinct preprocessing, analysis, and visualization workflows [46,82,85]. This results in differences in preprocessing, annotation, software versioning, and analytical implementation which can reduce reproducibility across platforms, introduce model bias, and reduce the utility of the model, as described in Section 2.4.

Various institutions have developed numerous computational tools for analysis and visualization of datasets. Xena, developed by the University of California Santa Cruz (UCSC), supports visualization, integration, and analysis of cancer genomics and the associated clinical data for both large public repositories and private datasets [86]. Similarly, OncoDB was created by researchers at the University of Illinois Chicago to analyze and visualize pan-cancer samples using interactive online analysis of TCGA and RNA-seq data from Genotype Tissue Expression (GTEx) [87]. TumorMap, another tool developed by UCSC, provides an interactive and easily interpretable portal to explore high-dimensional complex omics data by using similar underlying principles as Google Maps [88].

Tools built on Xena, such as UCSCXenaShiny v2, have continued to improve and gain functionality and usability, improve user interfaces, and add additional datasets, including those from other institutions [89]. OncoDB has also been upgraded to OncoDB 2.0, which improves analysis and visualization of TCGA and GTEx data by allowing simultaneous analysis of DNA and RNA somatic mutation data [90]. Table 4 lists representative tools selected to demonstrate commonly used approaches for accessing, integrating, analyzing, and visualizing cancer genomic and multi-omic data.

Continued maintenance and upgrades by both the originating and adoptive organizations remain the exception rather than the rule leading to a reproducibility risk. A survey of 2396 bioinformatics web tools from 2020 found that only 31% of tools remained continuously accessible over a period of 133 days, 48.4% were intermittently available, and 20.6% were never available at all. Recently published tools (less than 1 year old) were available for 90% of the test window, while 10-year-old tools were available 50% of the time [91]. A similar study of ~36,000 omics software resources found that 28% could not be reached at the published URL and 28% of a subset of 98 tools could not be installed at all. Cancer resources also suffer from deprecations like the Legacy ICGC 25K Data in 2024, requiring researchers to modify pipelines that depend on it [92]. These figures place a limitation on computational reproducibility, as pipelines with multiple published tools may be impossible to run with the failure of even a single tool. Fragmentation has a measurable cost that harmonized data does not address in this case, and alignment must extend to versioning, maintenance, and historical preservation of the analytical layers as well as the datasets. The analytical layer has the same alignment problem as the data, where tools differ in input representation and interfaces for exchange, so a pipeline developed from independently maintained tools inherits the representational and exchange misalignments even if the underlying datasets are harmonized.

Researchers often turn to custom pipelines to analyze information from a large Variety of databases using multi-tool methodologies. They deploy customizable scripting options such as Python or R libraries to assist with data analysis and visualization, such as Pandas, NumPy, ggplot2, Matplotlib and others. However, these options require extensive training and place additional demands on researchers when datasets use incompatible formats or interfaces. LLM-based coding agents such as ChatGPT and Claude can lower some technical barriers to developing these pipelines [83], but they do not resolve the interoperability issues in the underlying data. The code still requires validation for correctness and reproducibility.

**Table 4:** Selected visualization and analysis tools for cancer genomics data to support cancer drug discovery.

Tool	Developed By	Purpose
The Cancer Imaging Archive (TCIA) [93]	Frederick National Laboratory for Cancer Research University of Arkansas for Medical Sciences	Repository of de-identified medical images of cancer patients, freely available to the research community for analysis and algorithm development
The Cancer Proteome Atlas Portal (TCPA) [94,95]	The University of Texas MD Anderson Cancer Center	Access, visualization, and analysis of functional proteomics data from cancer cell lines and tumor samples, especially from TCGA
cBioPortal for Cancer Genomics [96,97,98]	Memorial Sloan Kettering Cancer Center Consortium of: Dana-Farber Cancer Institute Princess Margaret Cancer Centre Children’s Hospital of Philadelphia The Hyve Bilkent University	Exploration and visualization of large-scale multidimensional cancer genomic datasets including mutation, copy number, expression, and clinical data
TumorMap [88]	University of California Santa Cruz (UCSC)	Interactive portal enabling novice and expert computational biologists to explore high-dimensional complex omics data using Google Maps-inspired navigation
Xena [86]	UCSC	Visualization, integration, and analysis of cancer genomics and associated clinical data from both large public repositories and private datasets
UCSCXenaShiny v2 [89]	Central South University Sun Yat-sen University Cancer Center and other collaborating universities	Enhanced Shiny-based interface for Xena providing additional datasets, improved user interface, and expanded integrative analysis capabilities
MEXPRESS [99]	Ghent University	Visualization of TCGA expression, DNA methylation, and clinical data and their interrelationships to support epigenomic target identification
OncoDB 2.0 [87,90]	University of Illinois Chicago	Comprehensive platform for interactive pan-cancer omics analysis including gene expression, viral infection data, and simultaneous DNA and RNA somatic mutation visualization using TCGA and GTEx data
Gene Expression Profiling Interactive Analysis 3 (GEPIA3) [100]	Peking University Chongqing University Xi’an Jiaotong University	Web server for large-scale expression profiling and interactive analysis of TCGA and GTEx data, supporting differential expression, survival analysis, isoform quantification, and cancer subtype comparison. GEPIA3 builds on GEPIA1 and GEPIA2 and adds drug sensitivity information from 1000+ therapeutic compounds
Tumor Immune Estimation Resource 3.0 (TIMER3.0) [101]	Sichuan University Dana-Farber Cancer Institute Harvard T.H. Chan School of Public Health	Web resource for comprehensive analysis of tumor-infiltrating immune cells using multiple state-of-the-art algorithms applied to TCGA tumor profiles to explore tumor-immune interactions. TIMER3.0 builds on TIMER2.0 with 15 deconvolution algorithms for mouse-specific methods, immunotherapy response modules, and signature-based functional profiling

## 4. The Role of Big Data in Preclinical Cancer Drug Discovery

**Figure 2:**
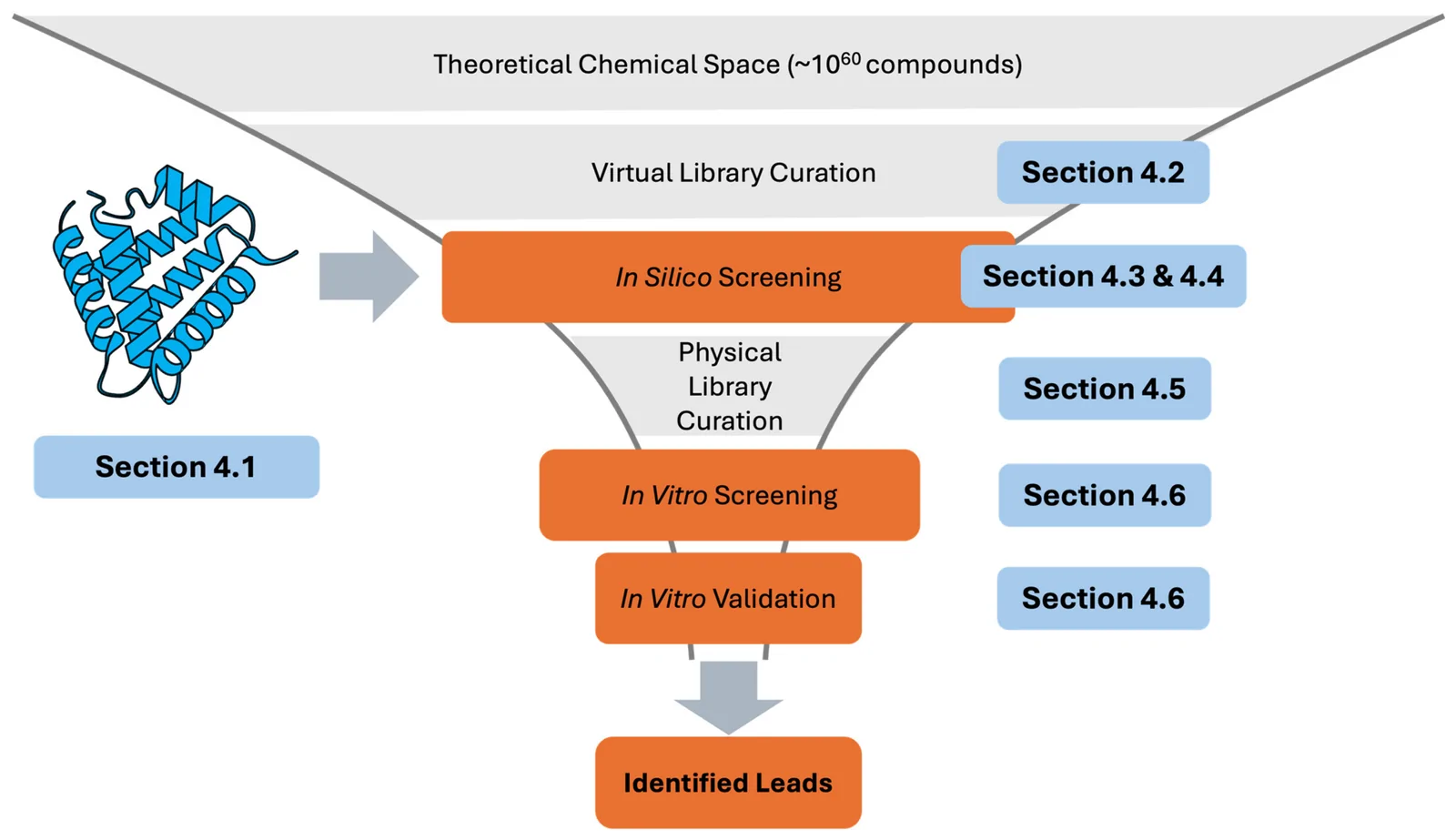
The narrowing of the chemical space towards identified lead through curation and screening processes are mapped to the corresponding sections of this review. Protein—attribution: Protein_colored icon by DBCLS https://togotv.dbcls.jp/en/pics.html accessed on 28 August 2026 is licensed under CC-BY 4.0 Unported https://creativecommons.org/licenses/by/4.0/.

Preclinical cancer drug discovery pursues two broad therapeutic modalities, small molecules and biologics, both of which are driven by large-scale, data-intensive screening. This section primarily focuses on small-molecule therapeutics with a brief section providing basic information on biologics. In each of the following subsections represented in Figure 2, the important question is not whether computational approaches replace experimental screening but rather how the data coordinate within and between the various processes, from target selection to library curation, prediction, synthesis, and screening.

### 4.1. Cancer Target Identification

The identification of an appropriate cancer target is one of the most important factors for deciding between in vitro and in silico methods. The nature of the candidates for screening is based on the cost and ease of production, validation, and complexity and is the driving factor in this decision [102]. For well-understood targets, pocket identification and ligand-based in silico screening can be optimal for large-scale screening, as discussed later in this section. However, some targets are not selected for study due to a lack of mechanistic detail and clarity to enable in silico screening and only become available with additional mechanistic insights. For example, transcriptional reprogramming and its implications in tumor progression and cancer heterogeneity have made it an attractive target for drug discovery, but the lack of a detailed mechanism behind transcriptional regulation hampered progress until the recent addition of drug discovery strategies leveraging HTS of gene expression signature changes [103]. In another case, Ras proteins long understood for their oncogenic potential, since their discovery in 1982, have only recently become a feasible drug target thanks to the discovery of a new allosteric binding pocket S-IIP [104,105].

As discussed later in Section 5.1, the big data behind EHRs and adverse event reporting can also reveal off-target activity or new indications for later target campaigns [90], emphasizing that target identification is not necessarily an isolated linear pipeline, but can instead be a circular process where preclinical development, clinical testing, and post-approval utilization inform subsequent discovery cycles. Additionally, large datasets such as TCGA can be used for the identification of pan-cancer targets such as HER2, allowing for anti-HER2 therapies to find candidacy in a variety of cancer types, including breast, colorectal, bladder, and lung cancers, among others [106]. In fact, within the HER2-positive landscape, PIK3CA mutations have been detected as another target of interest thanks to a meta-analysis of HER2 outcomes alongside TCGA mutational landscapes [92]. Likewise, the in silico tool DeepTarget identifies valuable cancer targets from the Cancer Dependency Map (Table 3) by integrating CRISPR knockout data as a proxy for drug inhibition with associated omic data and drug response profiles [93]. This approach also underpins the identification of synthetic lethal interactions, where loss of one gene becomes lethal only when coupled with an existing alteration.

Differences in experimental models, genomic annotations, preprocessing, and drug-response measurements must be accounted for and validated for relevance within the pooled data. Some studies can pull directly from databases like TCGA and the Cancer Dependency Map that already curate and link different experimental endpoints under intra-set definitions [91,93], but other projects combining multiple studies together must unify their different endpoints under a common calculation such as an odds ratio or hazard ratio in order to make an aggregated analysis [92].

### 4.2. Cancer-Specific Compound Library Curation

Focused library curation against specific targets can decrease the cost and time for initial screens and simultaneously improve hit relevance [107]. Target-specific libraries have emerged from non-guided screening against specific well-studied targets like kinases and G-protein coupled receptors (GPCRs) [108] with molecules from both existing collections or variations of efficacious scaffolds. However, the Volume of drug-like molecules, which ignores biologic constructs, is estimated at around 10^60^, making selecting and organizing compounds a greater challenge [109,110]. Data-driven, target-focused library curation is complicated by the Variability between tumors, heterogeneity within a tumor, and even emergent resistance over the course of treatment [111]. Consequently, researchers have explored oncology-focused libraries featuring additional layers of stratification based on cancer subtypes [111,112,113].

In addition to focused target-specific libraries, there are also chemical libraries built around high diversity, high similarity, or other shared characteristics. Diverse chemical libraries increase coverage of chemical space and the likelihood of bioactivity against a broad range of druggable targets, while high-similarity, combinatorial libraries tend to produce a multitude of bioactive hits or no activity [114]. Table 5 describes some common chemical libraries with notes about their subsets, size, and chemical availability.

Compound libraries follow similar informatics, namely with a chemical structural identifier alongside vendor and internal codes or annotated biological activities and synthetic schemes. The chemical identifiers of these libraries represent an area that benefits from the lessons of harmonized Vernacular. There are a multitude of chemical identifiers, such as SMILES strings, CAS numbers, or even common drug names, which serve to link to a registered chemical structure. However, there are published reports highlighting inconsistent or ambiguous structures from non-systematic identifiers, which poses a risk for merging and curating datasets from HMDB, ChEMBL, and PDB, among others [115]. Systematic identifiers, like MOL, SMILES, and IUPAC, are not invulnerable, and there can be inconsistencies in molecular representations between databases and even for a single compounds and its multiple systematic identifiers [116]. Attempts have been made towards harmonized Vernacular of molecular representation, such as the InChI string or a canonical SMILES input derived from InChI, to minimize ambiguity in linking chemical structure with associated properties and reactions needed for in vitro and in silico preclinical cancer drug discovery [117,118].

**Table 5:** Compound libraries and the vendors for both virtual libraries and real libraries.

Library	Size (Virtual/Physical)	Availability	Differentiating Features
Life Chemicals	V: 4.7 million P: ~540,000	In stock or synthesizable	Focused libraries Tangible/on-demand chemical space
ChemDiv [119]	V: 2 trillion P: 1.6 million (in stock) 12 million (managed	In stock, managed inventory, and synthesizable on demand	Focused libraries, ultra-large synthesizable space
ChemBridge [120]	P: >1.3 million	In-stock compounds	Focused screening subsets
Enzo Life Sciences [121]	P: >3000	In-stock compounds	FDA-approved drugs Repurposing and toxicity subsets
SPECS [122]	P: >350,000	In-stock compounds	Diverse. Natural Product, and ADMET filtered sets
ZINC [123]	V: >37 Billion (2D) >4.5B ready to dock (3D)	Readily purchasable compounds from other vendors	Vendor-linked chemicals, ready-to-dock subsets, and pre-annotated subsets
Enamine REAL [124]	V: >94.5 Billion	Synthesizable on demand	Diverse. Reaction-validated pre-annotated bioactivity
Asinex [125]	P: >500,000	In-stock compounds	3D, natural product like, and targeted scaffolds
Maybridge [126]	P: >53,000	In-stock compounds	Diverse. Fragmented screening collections and drug-like compounds
ChEMBL [77]	V: >2.9 million	No availability. External vendors only	Literature curation of compounds with bioactivity data
SCUBIDOO [127]	V: >21 million	Virtual compounds designed for synthesizability	Annotated with necessary synthesis steps
SAVI [128]	V: >1.7 billion	Virtual compounds, but are synthesizable	Annotated with necessary synthesis steps and predicted ADMET

### 4.3. Virtual Ligand Screening

Hit identification by experimental assays is time-consuming, expensive, and prone to false positives [66,129]. In silico modeling, which has become more popular, addresses this by performing virtual ligand screens (VLS) to advance more promising candidates to experimental testing [130,131]. VLS can investigate questions that conventional experimentation cannot, drawing on resources such as experimentally-solved, agonist-bound androgen receptors [130,132]. Focused library curation occurs on customized virtual libraries (Table 5), evaluating compounds that would otherwise be too expensive or difficult to synthesize [130]. In oncology, where the biological target is multi-omic and complex, VLS is most useful against a structurally well-defined target whose ligand-receptor interactions can be modeled with sufficient confidence.

**Figure 3:**
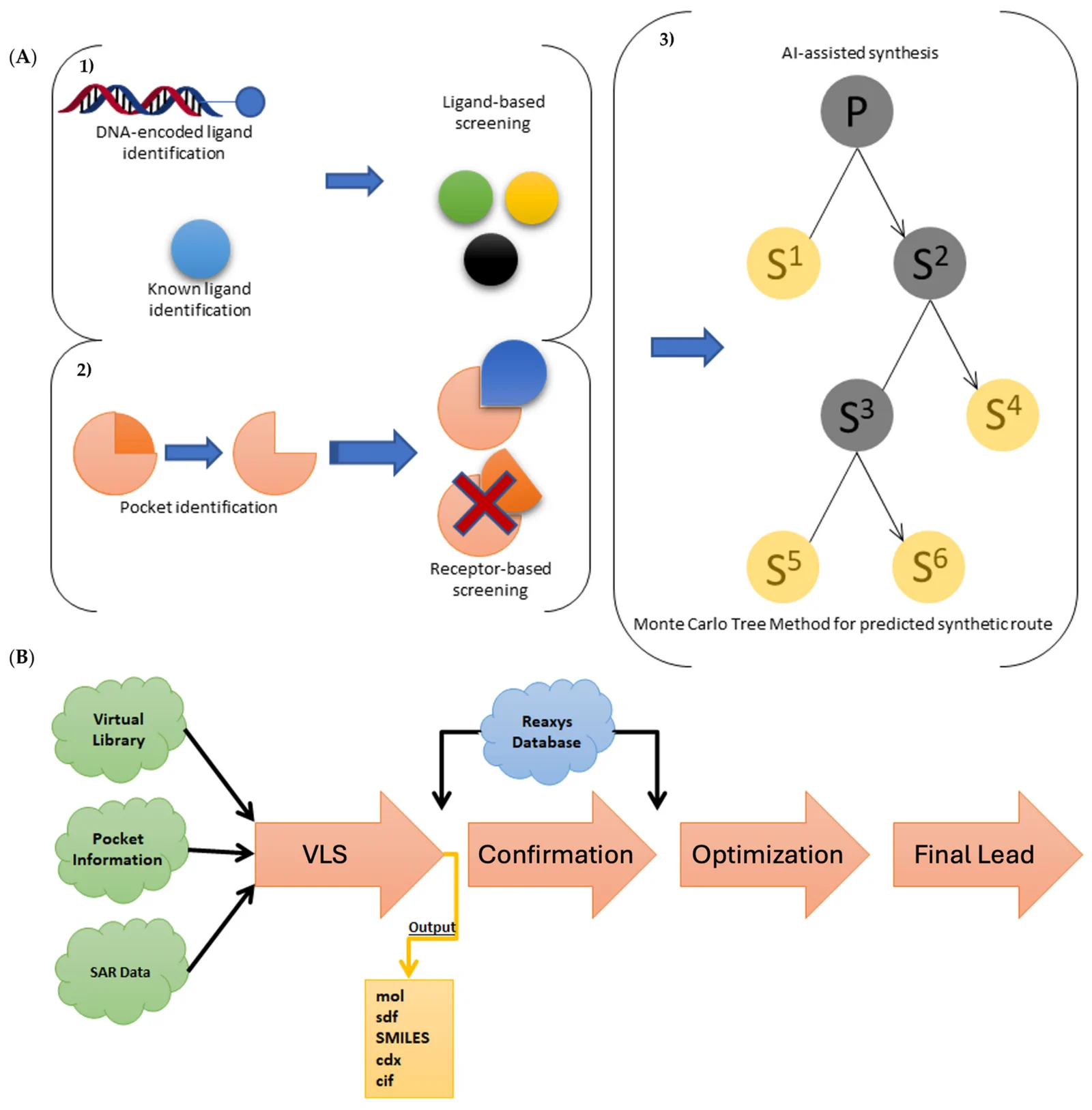
(**A**) Workflow for in silico preclinical cancer drug discovery. Lead identification can occur using two different methods: (1) DNA-encoded ligands to determine ligands with the greatest affinity with the utilization of known ligands as a training set leading to a ligand-based screen and (2) structure-based identification of the binding pocket that docks virtual libraries into the pocket with X denoting a binding mismatch. Both these methods lead to (3), AI-assisted backwards synthesis known as retrosynthesis, such as the Monte Carlo Tree Method with multiple branches. The reactions continue to add branches until the starting material is commercially purchasable (yellow), while the nodes (gray) determine unpurchasable material, branching off from the finalized product (P) based on the reactants (S). (**B**) Virtual Ligand Screening (VLS) schematic with data compiled from virtual libraries, receptor pockets, and structure-activity relationship (SAR) of active compounds. After VLS, reaction databases such as Reaxys can be used for synthetic predictions leading to optimization and lead compounds for further testing and verification [143].

VLS generates large Volumes of data at high Velocity through two complementary approaches, compound-based (ligand-based) and receptor-based (structure-based) [133,134]. Both require computational analyses of virtual libraries and an understanding of the target of interest (Figure 3B). Compound-based VLS (CBVLS) models require a compilation of known inhibitors, typically through quantitative structure–activity relationships (QSAR), to identify new ligands sharing similar features [133]. These models are trained on one-, two-, and three-dimensional descriptors of the known ligands and use the QSAR and quantitative structure property relationship data to determine the best pharmacophore scaffolds and substituents for activity. Then, they search virtual libraries for compounds containing similar pharmacophores [135], using the Tanimoto coefficient to quantify the similarities of features between the compounds [136].

A principal limitation of CBVLS is that the limited amount of available data results in the output scaffolds being similar to previously identified compounds, hindering the novelty and value of the compound [122]. Additionally, reviews covering QSAR studies are increasingly concerned over the state of QSAR models as disconnected, isolated, customized pipelines, leading to an inability to harmonize or even reproduce their model findings [137,138,139]. As a result, researchers have also taken steps to harmonize the QSAR model space by focusing on standardizing the semantics guiding their desired QSAR molecular descriptors and underlying calculation methods [139]. Other studies have also worked on providing a hierarchical framework to organize the representation of chemical identifiers with their associated structures, properties, and bioactivities [138]. It should be noted that within these example frameworks, the semantics of their molecular descriptor calculations are still evolving, which raises another concern over the long-term semantic harmonization needed to promote inter-dataset communication [138,139].

The other face of virtual compound discovery, receptor-based VLS (RBVLS), docks a virtual library of compounds into the known binding pocket [134]. This receptor-based approach is the most widely used in silico technique, reflecting the sustained study of docking between compounds and active sites [66]. It triages by escalating from a rapid two-dimensional analysis of the full library to a three-dimensional analysis of the most promising candidates. RBVLS requires knowledge of the protein receptor’s binding mechanism before progressing to the ligand–receptor interactions. Flexible docking accommodates the conformational changes in both the ligand and receptor, and molecular dynamics calculations assess the stability of the ligand–receptor binding to improve confidence in the predicted binding affinity [140,141]. The available software packages (Table 6) are key parts of RBVLS and apply scoring functions grounded in physics-based, empirical, knowledge-based, machine-learning, and artificial intelligence-based algorithms [142].

Docking programs span across both physics-based and machine-learning-based methods, so various studies have emerged to benchmark these machine-learning-based methods to assess their Veracity to physics-based methods. An initial benchmark study in 2023 demonstrated that the <2 Å root-mean-square deviation (RMSD) between predicted and experimental ligands was an insufficient benchmark and included additional benchmarks assessing valid intramolecular geometry and non-clashing intermolecular distances [144]. The inclusion of valid molecular geometry in their PoseBusters benchmark revealed that all deep-learning docking methods (DeepDock, Uni-Mol, DiffDock, EquiBind, TankBind) had a dramatic drop in valid dockings compared to just assessing RMSD between predicted and experimental ligands, whereas their non-ML physics counterparts AutoDock Vina and CCDC GOLD were relatively unaffected, having similar percentages of valid dockings with and without valid molecular geometry penalties. A more recent study undermines this claim by pointing out that the PoseBusters benchmark lacked cross-linking assessment, which is a more practical consideration when using VLS to explore novel compounds compared to self-docking of the same co-crystallized ligand. With the inclusion of cross-docking with additional energy minimization, ML-based methods like Uni-Mol, SurfDock, and DiffDock retained superior success rates in docking compared to traditional physics-based methods (AutoDock Vina, Glide, Discovery Studio) [145].

While Veracity is an ongoing concern to validate machine-learning methods against physics-based methods, the principles of semantic alignment are studied at all elements of docking [146]. One group noted that arbitrary decisions are made when preparing a protein PDB for docking, such as the use of homology and generative modeling, preparing a 3D ligand structure from its chemical identifier, and which scoring functions, like RMSD or ROC-AUC, are utilized [146]. As a result, PLATE-VS has sought to curate and cross-communicate protein, activity, and ligand inputs using PLINDER structures, UniProt protein identifiers, and annotated bioactivities from ChEMBL, all while including multiple methods of machine-learning and physics-based docking [147]. However, much like DockBench, DockM8, PoseX, and PoseBusters, the comparison of multiple docking methods only acts as a consensus model rather than a singular harmonized system, meaning the actual docking methodologies are still a subject to address as part of the Vernacular principle [54,144,148,149].

The defining feature of RBVLS is the use and requirement of a three-dimensional structure of the target, historically obtained from the Protein Data Bank (PDB), which aggregates experimentally determined structures from X-ray crystallography and related methods and has increased in size approximately 10% annually [79,150]. However, as experimental structures cannot keep pace with the number of candidate targets that expanding multi-omic datasets generate, deep learning structure prediction has become central to meet this Volume of demand. AlphaFold2 transformed the field by achieving near-experimental accuracy at the 14th Critical Assessment of the Protein Structure Prediction (CASP14), predicting structures from a sequence with a high degree of fidelity [151,152], providing the ability to obtain a large Volume of protein structures at a high Velocity. Additionally, OpenFold, an open-source, retrainable implementation of AlphaFold2, reproduced AlphaFold2’s accuracy and provided the training code and data, allowing the model to be retrained for new tasks such as protein–ligand complex prediction while remaining robust even with limited training data [153]. Regardless of the method for sourcing the target’s three-dimensional structure, pocket identification commences to find the target’s pocket of interest using geometric and energetic algorithms, such as atom flattening (closing pocket and determining as set of spheres), discrete flow analysis, past α-shapes, and a combination of methods combined with statistical analysis [135,154,155,156,157]. PyMol and JMOL can be used to visualize the predicted and experimental structures for interactions.

However, predicted structures are not interchangeable with experimentally determined structures or testing via in vitro screening. In a docking-based virtual-screening benchmark, AlphaFold models have consistently underperformed experimental PDB structures in docking programs, because they are generated without the bound ligands, ions, or cofactors which can substantially affect docking outcomes [158]. Even when PDB models were stripped of their cofactors, ions, waters, ligands, and metals akin to their AlphaFold counterparts, the crystal structures always performed higher, with enrichment factors of 20.5 on average compared to the AlphaFold enrichment factors of 8.8 on average [158]. Another limitation of these prediction models is that their underlying training set biases can affect the conformation in the outputted prediction [148]. This is particularly important in oncology, as most experimentally determined kinase structures are enriched for active-like conformations (DFG-in) and thus artificially limit screening towards DFG-in inhibitors instead of diversely exploring DFG-in, DFG-out, and even allosteric inhibitors [148]. Newer prediction models like AlphaFold2 can mitigate this issue through a partial recovery of alternative kinase conformations while improving docking and VLS performance [159]. This gap is also being addressed with newer models such as AlphaFold3’s direct prediction of protein–ligand complexes with holo (liganded) models built around the active ligand outperforming apo (unliganded) predictions in screening [160].

**Table 6:** Docking software for virtual screening.

Docking Software	Developed By	Access	Purpose
AutoDock Vina [161]	Scripps Research Institute	Free, Open source	Rigid and flexible molecular docking. Widely used with macrocycles, batch/multi-ligand docking
DOCK [162]	University of California, San Francisco	Free	Rigid and flexible molecular docking
Glide [163]	Schrodinger	License Required	Rigid and flexible molecular docking with different modes: high-throughput virtual screening, standard, and extra precision
ICM-Pro [164]	Molsoft LLC	License Required	Rigid and flexible molecular docking with protein structure analysis, pocket finder, structure prediction, interactive ligand editing, and protein structure prediction
CABS-dock [165]	University of Warsaw	Free	Flexible docking peptides to protein without prior binding-site knowledge
GNINA [166]	University of Pittsburgh	Free, Open source	Convolutional Neural Network scoring-based molecular docking
DiffDock [167]	Massachusetts Institute of Technology	Free, Open source	Diffusion-based generative model for blind docking with confidence estimates
KarmaDock [168]	Shanghai Institute of Materia Medica, CAS	Free, Open Source	Deep learning docking optimized for large-library screening
SurfDock [169]	Shanghai Institute of Materia Medica and Zhejiang University	Free, Open Source	Surface-informed equivariant diffusion model for protein–ligand complex prediction and virtual screening

### 4.4. Unified, Iterative Library Curation and In Silico Screening

The rapid growth of VLS has challenged the capabilities of computational tools to brute-force the docking. This is untenable as many make-on-demand libraries have expanded to billions of compounds. The sheer Volume of possible compounds makes it impossible to generate sufficient Velocity of results within practical computational resource usage. As a result, combinations of compound management and VLS functions are appearing as unified screening tools to lower the computational resources used in navigating large libraries. These programs only need to screen subsets of chemical libraries and use iterative cycles of evaluation and compilation to generate a final collection of high-scoring hits without exhaustively screening the entire chemical space. The specifics of each program, such as the source library, scoring method, and iteration method will differ, with each program presented below in Table 7. ML tools, rather than measurements using experimental assays, have also increasingly been used to predict the toxicity and ADMET properties [128,129]. As an example, the end-to-end ATOM Modeling Pipeline, built on the DeepChem library, trains models for pharmacokinetic and safety properties from historical drug discovery data [130]. These pipelines are now being extended to repurposing FDA-approved drugs, for example, dual wild-type and exon-20-mutant HER2 inhibitors [131]. Recent reviews catalog similar docking, QSAR, free-energy, and generative-design modules across the cancer subtype space [132]. These approaches use the Volume and Velocity of expanding chemical libraries to prioritize smaller candidate sets, but their Value remains dependent on the Veracity of the training data, structural models, and subsequent experimental validation.

While VLS has grown with increasing Volume and Velocity, the Veracity of hit compounds still requires experimental verification by in vitro assays. Hantz and Lindert demonstrated that selecting docking receptors for their ability to enrich known activities markedly improves true-positive rates and validated the approach experimentally on cancer-related targets. They identified 22 novel inhibitors by scanning a subset of five diverse cancer-related disease targets, with the inhibitors spanning the low-micromolar to nanomolar range. The most potent was an EGFR inhibitor with an IC50 = 7.96 nM [170]. VLS’s advantage is therefore real but conditional, and it is best used to complement experimental screening rather than being used as a full replacement. This verification depends on being able to synthesize the compounds, as discussed in the next section. This computational-to-experimental cycle reflects that increasing data Volume and computational Velocity can reduce the experimental search space, but translational Value is dependent on Veracity and validation at each subsequent stage of the drug discovery pipeline.

**Table 7:** Iterative compound library screening and generation software accessible to researchers.

Screening Platform	Library Source	Iteration Methodology (for Next Cycle)	Scoring Methodology	Location
AlvaBuilder [171]	User-provided	Structural recombination and mutation of high-score candidates generates next population	QSAR and structural similarity scoring	https://www.alvascience.com/alvabuilder/ (Accessed on 27 July 2026)
HASTEN [172]	User-provided	ML model trained on docked subset to select high-score candidates for next docking subset	Docking scoring via FRED or Glide	https://github.com/TuomoKalliokoski/HASTEN (Accessed on 27 July 2026)
REvoLd [173]	Enamine REAL	Structural recombination and mutation of both high- and low-score candidates generates next population	Docking score via RosettaLigand	https://docs.rosettacommons.org/docs/latest/revold (Accessed on 27 July 2026)
SpaceGA [174]	User-provided	Structural recombination and mutation of high-score candidates generates next population	Docking score via AutoDock-GPU	https://github.com/lmoesgaard/SpaceGA (Accessed on 27 July 2026)
V-SYNTHES [175]	Enamine REAL	Addition of chemical group “synthons” diversifies high-score candidates for next docking subset	Docking score via ICM-Pro	https://github.com/katritchlab/V-SYNTHES (Accessed on 27 July 2026)

### 4.5. Chemical Synthesis and Generative Design with Machine Learning

Following hit identification, a synthesis route must be identified or designed (Figure 3), a step where human expertise and machine-extracted knowledge can be complementary [176,177]. DNA-encoded libraries (Figure 3A) assist in this manner, as each compound carries a DNA barcode identifying specific reactants and building blocks to produce the compound [131]. Additionally, algorithms are trained on large reaction libraries, such as Reaxys^®^ and SciFinder^®^, which catalog tens of millions of reactions, substances, and bioactivity records to construct a reaction route and optimal conditions (Table 8 provides further examples). Veracity is the central constraint because reaction data are inconsistent in quality, resulting in the generation of overly simplistic models and incomplete reaction sets. As a result, it can be a challenge to obtain feasible chemical routes on a given scale.

These models typically output a retrosynthetic scheme. In 2018, Segler et al. combined Reaxys^®^ with three neural networks for retrosynthesis guided by Monte Carlo tree search (MCTS) to map synthetic routes [178]. Similar models provide the necessary purchasable reactants to obtain the desired product and a possible product based upon unknown starting materials and reactants [177]. In the past few years, the field has shifted from template and MCTS-based approaches toward transformer-based AI architectures that recast synthesis as a sequence-to-sequence translation between product and precursor SMILES strings. Several paradigms have been established for the following: (a) forward reaction prediction to infer products from given reactants [179]; (b) template-free retrosynthesis models, which generate precursors directly and correct chemically invalid outputs [180]; and (c) approaches that more closely mirror a chemist’s logic by decomposing a target into smaller synthons before reconstructing the viable reactants [181]; thus, an impressive array of computational tools is currently at the disposal of the modern medicinal chemist [182,183]. However, the reliance on SMILES in these transformer-based systems makes identifying the reaction center at each stage difficult, in part since multiple SMILES entries are valid for the same structure, creating a “many-to-many” problem that further obscures where bonds are formed and broken [184].

ML and human expert knowledge each have limitations but implemented together provide potent synergy. The foundational ML expert-system is Chematica, now commercialized as Synthia, which encodes roughly 50,000 reaction rules spanning common and advanced methodologies [185]. Badowski et al. trained a model with rules modified to exclude protection and deprotection steps in order to afford access to more complex structures [176]. The retrosynthetic tree was built with ~100 branches per step, pruning unpromising paths until the route resolved to one using commercially available starting materials [33,186]. Both approaches are constrained by the Volume and Veracity of reaction data available to train the models. In fact, the diversity of the training set greatly affects single-step retrosynthetic models like those of MEGAN, LocalRetro, and RootAligned, meaning Synthia’s foundation on curated reaction rules also makes it vulnerable to these biases [187]. Here, transfer learning offers a partial remedy, using generalizable knowledge from larger related datasets to train models where data are sparse [188], a recurring constraint across the preclinical pipeline.

Generative models design molecules de novo rather than retrieving them from searches of existing libraries. Zhavoronkov et al. have developed a model called Generative Tensorial Reinforcement Learning (GENTRL), which nominated potent DDR1 kinase inhibitors after training on multiple datasets and libraries like ZINC compounds, known inhibitors, patent data, and 3D structures [186]. The field has since expanded from reinforcement learning approaches to transformer and diffusion models [33]. A persistent limitation is synthesizability, because generative models often propose molecules that cannot be synthesized, precluding experimental validation. Software such as SyntheMol addresses this problem by assembling candidates from commercially available precursors using validated reactions to ensure that every generated molecule has a feasible synthetic pathway [189]. Thus, molecular generation does not establish Value because generated compounds must remain synthetically accessible and ultimately be validated experimentally.

Robots designed to optimize strings of reactions increasingly perform the synthesis with automated analytical tools [190]. At AbbVie Inc., Baranczak et al. built an integrated platform for small molecules through synthesis, purification, quantitation, dissolution, and testing. They validated this platform with a 24–36 h turnaround against the embryonic ectoderm development subunit of polycomb repressive complex 2, a chromatin-regulatory complex implicated in several cancers [191]. Eli Lilly’s Idea2Data offers a further example of linking in silico design, synthesis, characterization, purification, and biological testing [192]. In a more recent example, Novartis MicroCycle coupled microscale synthesis, automated purification, and biochemical and cellular assays, feeding the results back to active-learning models that select the next round of compounds [193]. Such efforts are important avatars of a future when automated laboratories operate iterative cycles of drug discovery with human oversight but minimal intervention.

With the advent of agentic large language models as synthesis copilots, several systems combine cheminformatics and reaction prediction tools to design synthetic routes, such as ChemCrow [194]. Coscientist, a multiagent system driven by GPT-4, combines LLMs to design and run reactions robotically [195]. Recently, Multiple Optimized Specialists for AI-assisted Chemical Prediction (MOSAIC) has included known literature reaction protocols to create reproducible, executable procedures and achieved a 71% success rate [196]. These examples emphasize the distinction between generation and validation, where an AI-proposed route is only of Value when its feasibility and reproducibility can be demonstrated experimentally. The same predictive and generative approaches extend to biologics, which are discussed in Section 4.7.

**Table 8:** A representative list of tools to assist with synthetic feasibility.

Synthesis Tool	Developed By	Purpose
Reaxys^®^ [143]	Elsevier	Searchable collection of chemistry literature and bioactivity data; Retrosynthetic predictor Currently has 69 million reactions; 160 million substances; 48 million bioactivity datapoints; 43,000 biological targets; 119 million documents
SciFinder [197]	CAS	Searchable collection of chemistry literature with a reaction and bioactivity database
Deep neural network retrosynthesis (3N-MCTS) [178]	Westfalische Wilhelms-Universitat	AI retrosynthetic route planning with Monte Carlo tree search over neural networks
ChemFormer [198]	AstraZeneca	Pre-trained SMILES transformer for forward reaction and single-step retrosynthesis prediction
RAScore [199]	AstraZeneca	ML classifier tests synthesizability for large libraries of compounds
Generative tensorial reinforcement learning (GENTRL) [186]	Insilico Medicine	De novo drug design to optimize synthesis, novelty, and biological activity
IDOLpro [200]	Sandbox AQ	Guided diffusion generative model optimizing for binding affinity and synthetic accessibility around structure-based design
Integrated synthesis-purification-bioassay system [191]	AbbVie Inc.	Automation of synthesis, purification, quantitation, dissolution, and testing of molecules
Idea2Data [192]	Eli Lilly	Processing of billions of virtual molecules, to determine most likely synthesizable on an automated system for biological testing

### 4.6. High Throughput and High-Content Screening in Oncology

Automation of assays has become integral in drug discovery thanks to enabling high Volume, high Velocity data acquisition in the form of large-scale parallel testing high-throughput screening (HTS) and high-content screening (HCS). HTS is designed as a miniaturized biochemical or biological assay optimized for fast, automated liquid handling and processing of large numbers of compounds in 96-, 384-, and 1536-well formats [201,202]. To facilitate public screening, the NIH established the MLPCN, a library of more than 330,000 small molecules screened across nine academic centers using target-based endpoint assays and analyses of different protein expression and cellular viability [66,129]. These HTS assays need to be validated through a pilot screen to ensure relevant, consistent biological endpoints and thresholds compared to prior historical data [66,129]. Primary screens identify hits passing the selected threshold, at which point they undergo additional confirmation assays to eliminate false positives [66]. Surviving hits then undergo multi-point titration to quantify half-maximal inhibitory concentration (IC_50_) for further development such as dose-profile therapeutic window and preliminary absorption, distribution, metabolism, excretion, and toxicity (ADMET) characterization [203]. This HTS progression provides experimental validation of identified compounds from in silico screen results and provides data back to the models for improvement in subsequent campaigns [193]. Screening hits nonetheless also must be verified with dose-response and further assays beyond HTS.

Similar to VLS, each successive step increases complexity, either through additional assay interrogation by adding more data points (confirmation and titration assays) or data fields (HCS) or through more biologically complex models. Oncology screening campaigns are increasingly utilizing more complex biological models and are moving from two-dimensional cell lines to more disease-relevant models like three-dimensional cell line spheroid screens and patient-derived organoid (PDO) models for glioma stem cells and non-small-cell lung cancer, respectively [204,205]. A 2016 study tested the activity of a pancreatic cancer cell line (PANC-1) as both 2D culture and 3D spheroids against 1912 oncology compounds at 11 different concentrations. The screening results showed that at the maximum dosage, 3D spheroids were 40% less active than their 2D counterparts and had IC_50_ values 10-fold higher in four of the therapeutic classes [206]. In another study in ovarian cancer models, Carboplatin response was tested in six 2D cell lines, the same six 3D spheroid lines, and five 3D ex vivo tumors, and their responses were compared against in vivo mouse xenograft models. Three out of six 2D cell lines, four out of six spheroid lines, and five out of five of the ex vivo tumors correlated in response to the mouse models [207]. Both these studies show a general direction rather than direct evidence of better in vivo response prediction with increasing complexity of biological models.

PDOs extend this progression by adding patient-specific biology, and their predictive Value has been quantified against clinical outcome. In a systematic review of 17 oncological studies for tumor response, the PDO establishment range was reported by 12 studies and ranged from 31% to 90% with a pooled establishment rate of 68.5% and required between 10 to 34 days. The pooled sensitivity and specificity values for clinical response were estimated to be 0.81 and 0.74, respectively [208]. Heterogeneity, tumor type and reported pooled numbers limit interpretation and utility. Conventional two-dimensional screening persists not because better models are not available, but for wider access and faster results.

These more complex models generate more biologically relevant information than the typical HTS campaign with its generally single endpoint readouts, but they also demand new types of data be captured [204,205,209,210]. HCS meets this need by using automated microscopy to provide multifaceted hit classification through cellular phenotypes such as morphology, growth, extracellular matrix reconfiguration, and other complex organoid responses rather than the simple quantitative readouts of HTS [209,210]. However, HCS reduces screening throughput by 1 to 2 orders of magnitude compared to HTS due to the rate limits imposed by image acquisition and processing, though hardware and software improvements with instrumentation, parallelization, and machine learning of cellular features are helping address these limits [211].

The creation of harmonized, inter-dataset HTS is the subject of several shared databases for biological activity like PubChem, Comparative Toxicogenomics Database (CTDbase), BindingDB, and ChEMBL [212]. While each database has their own internal identifier like PubChem’s CID or CTDbase’s MeSH, they also incorporate Vernacular by providing a shared format as SMILES, InChIKey, or InChI, albeit as a supplement rather than the primary identifier [212,213,214]. The chemical identifiers are independent of instrument, plate format, and institution, so deposition and cross-dataset aggregation proceed automatically upon registration of a compound.

While HTS data can be harmonized through the chemical identifiers, HCS by contrast remains deeply fragmented. HCS fragmentation is present at nearly all stages, from the imaging technology to the image file formats to even the annotations and metadata underlying HCS runs. The fragmentation begins at the instrument, as each imaging platform writes its own proprietary file format and acquisition metadata. The output is tied to the vendor that produced the microscope. Researchers bridge the gap with custom code rather than shared standards, converting the formats and rewriting metadata prior to analysis. While there is still no universal framework, there are several initiatives to harmonize and standardize HCS, such as Open Microscopy Environment (OME), which defines OME-TIFF as a standard image format or OME-NGFF as a cloud-native format [215,216,217,218]. Conversion nonetheless remains a translation step rather than a common representation, and segmentation methods, feature definitions, and experimental annotation are unaligned [219]. This can be seen in large image repositories. The BioImage Archive, established at EMBL-EBI in 2019 as a counterpart to resources such as PubChem, as of 2022 holds 1200 datasets and over 1.5 petabytes of data [220]. Approximately 75% of the core collection is stored as TIFF, but more than 30 distinct file types remain in use, and this diversity remains a challenge to visualization without format conversions [220]. Thus, centralized deposition can improve data accessibility without ensuring that independently generated imaging datasets can be readily combined.

HTS and HCS approaches differ across all dimensions of Vernacular. Exchange alignment has been solved for both with standardized deposition with PubChem [221]. Bio-Formats and OME-TIFF allow microscopy images to move between systems [216]. Representational alignment is stronger for HTS because of the chemical identifiers providing a common anchor point across instruments and databases, while HCS remains divided among image formats and acquisition metadata. Semantic alignment is also difficult for HCS because derived features such as morphology and depend on segmentation and feature extraction. Finally, governance becomes increasingly important as models move from immortalized cell lines towards patient-derived materials for consent and use authorizations. Thus, greater HCS Volume does not increase shared Vernacular.

An inverse relationship between data Value and Volume/Velocity characterizes HTS, HCS, and in silico screening approaches. HTS maximizes Volume and Velocity of data with the single endpoint readouts, while HCS trades the Volume for the Variety of data through the phenotypic depth. In silico screening, on the other hand, is capable of very high Volume and Velocities of data but is limited by the Veracity of the underlying data, structural assumptions, and models used for prediction [130,131]. Rather than treating the three approaches as competitors, these approaches should be used complementarily by triaging from in silico (prioritizing candidates) to HTS and finally to HCS to provide experimental results providing validation and additional data for computational models.

### 4.7. AI-Driven Biologics Discovery

The same computational pipeline structure that underpins small-molecule oncological drug discovery also extends to biologics. The workflow sequence remains structure prediction, library and sequence management, screening, and de novo design but focuses on peptides, antibodies, and other biologicals. Target structure prediction is central as is the case for small molecule drug design, with AlphaFold3 capable of modeling biomolecular complexes, including antibody-antigen interfaces to predict epitopes, the biological equivalent of pocket identification [222]. Software, such as RFDiffusion, generates accurate de novo antibody designs [223], and protein language models elucidate binding affinity directly from the primary structure [224]. These foundation model approaches increasingly frame antibody engineering as a generative problem rather than a screening problem.

Although screening retains a high-throughput experimental core, it prioritizes development through model triage for aggregation, expression, and immune liabilities before synthesis. Protein language models can triage large antibody libraries for candidates with sequence features associated with clinical acceptance and synthesizability [225]. This compresses the design build test loop and is critically important for oncologic drugs that often rely on the interruption of specific pathways in the cell. As is the case for small molecule drug discovery, data sparsity and data sharing (Volume and Vernacular) limitations remain challenges. For biologics, increasing data Volume is therefore most useful when sequence, structure, activity, and synthesizability can be integrated with sufficient Veracity to support model training and experimental validation.

## 5. Cancer Clinical Trials: Drug Utilization and Artificial Intelligence

Clinical trials are the most time-consuming and expensive portions of drug discovery. Oncologic drug development times and costs vary considerably, with Sertkaya et al. placing average phase I, II, and III trials at 10.8 years and 670.8 million USD (inclusive of failed programs and capital costs), with less than a 5% chance of making it through all three clinical trials [14].

Section 4 described a preclinical pipeline in which accumulated data informs the next experimental decisions. Evidence that this improvement persists across the preclinical to clinical junction remains limited. Most projects with AI lead drug discovery remain preclinical, and a disease-agnostic cohort analysis found that 75 have been entered into clinical trials since 2015, with 67 still in trials as of 2023, mostly in phase I and phase II. AI-discovered molecules complete phase I with higher success rates (approaching 90%) compared to historical success rates (40–65%), while phase II results remain consistent with historical averages of approximately 40% [226].

The improvement in phase I vs. phase II remains contested. Bender et al. note that most AI-derived programs address established disease biology and chemistry lower in the probability of encountering safety, tolerance, and pharmacokinetic problems. Phase I success in established biology does not translate to therapeutic efficacy in phase II. Reducing failure rates at phase II leads to reduction in capitalized cost per success. Despite this, the majority of AI in drug discovery still focuses on preclinical hit discovery due to the availability of labeled data [227]. Patient stratification based on biomarkers in clinical trials and drug development significantly reduced the capitalized cost per successful launch by approximately half throughout the drug discovery pipeline up to phase III [227].

Several obstacles account for the gap with low shared Vernacular across trial documentation, fragmentation and restricted access to patient data, heterogeneous populations, and regulatory requirements for approval. These constraints concern whether information generated at one stage can be interpreted, validated, and acted upon at the next stage rather than the Volume of existing information. Nevertheless, computational approaches increasingly leverage information from past trials to support outcome prediction, patient selection, dose optimization, adaptive trial design, and other aspects of clinical development [228].

### 5.1. Predicting Clinical Trial Outcomes

The goal is to instrumentalize the accumulated Volume of prior clinical trial data to predict the success of an oncologic drug and to reduce time and cost to market. Both ML and LLM approaches have been evaluated for this purpose [42,229,230,231]. One of the main prediction tools is the Hierarchical Interaction Network (HINT), which takes in complex interactions of multi-modal clinical trial outcome data using a comprehensive Volume of clinical trial data curated into the Trial Outcome Prediction (TOP) dataset to model the interactions between the trial components. This model uses graph training to generate the predictive scores of clinical trial success. Harmonic mean scores of total vs. correct predictions of phase I, II, and III trial successes were 0.665, 0.620, and 0.847, respectively, and ranked among the better predictors compared to other machine learning methods like DeepEnroll and COMPOSE [230].

Aliper et al. designed the inClinico platform, another model for prospective clinical trial prediction, which predicts phase II to phase III success [229]. Their model uses transformer-based ML models combined with multi-modal datasets including omics, trial design, small-molecule libraries, and biological targets chosen from publications and data sources using GPT-3.5. Trained on over 55,000 trials initiated prior to 2018 from ClinicalTrials.gov, the model attained a receiver operating characteristic area under the curve (ROC-AUC) of 0.882 for phase II to III transition [229]. Additionally, the authors found that inClinico’s best predictive signal was the biological target of interest (0.841 ROC-AUC) instead of the trial design (0.582 ROC-AUC), supporting the assessment that the selection of the target biology is the more important parameter. Prospective predictions reached 79% accuracy for 17 trials in forecast of phase II to phase III transitions published between 2020 and 2022 [229]. Although prospective prediction strengthens the evidence beyond retrospective benchmarking, seventeen trials cannot establish generalizability and can be subject to survivor bias and reinforcement loops that favor previously successful areas, potentially reducing the biological novelty of the trials they promote [227].

Both tools mentioned in this subsection are disease-agnostic. As mentioned at the start of this section, oncologic drugs generally have low chances of successfully emerging from clinical trials. In a recent pre-print, HINT was compared with LLMs for predicting cancer trial outcomes underperformed in this role, with a balanced accuracy of 0.525 yet poor oncology sensitivity of 0.246, despite oncologic drugs being the largest portion of both their dataset and of TOP. Newer models such as GPT-4o or Llama3 have shown overall higher sensitivity (1 and 0.964), but this is tempered by a dramatically lower specificity (0.214 and 0.092, respectively), suggesting a need to improve negative predictions compared to HINT’s specificity performance of 0.473 as the highest among all models [42]. Thus, the comparison does not establish a clear winner. Instead, it demonstrates that aggregate performance metrics can conceal clinically important model failure modes. Oncology trials fail for reasons recorded in the documentation and correlative studies rather than in metadata.

### 5.2. Informing Clinical Trials

Where predicting clinical trial results uses data trained on prior trials, AI and big data have helped improved the methodologies of the trials themselves using previous data. Dose selection is an area of active reform. Oncologic dosing has historically been set at the maximum tolerated dose (MTD), which suits cytotoxic chemotherapy but is poorly suited for targeted agents where efficacy plateaus below the toxicity ceiling. In recognition of this, the FDA Oncology Center of Excellence established Project Optimus to optimize dosing throughout oncology drug development. Sotorasib, a KRAS G12C inhibitor enabled by the switch-II pocket (Section 4.1), illustrates the necessity for Project Optimus. The FDA initially approved Sotorasib at 960 mg daily, but a subsequent randomized dose comparison showed a response at 240 mg, although the arms were not formally tested against each other [232,233]. Despite the recent establishment of Project Optimus, significant increases in dose-optimization plans have been observed, with 30% of all trials including dose-optimization and an increase in the adoption of more complex methodologies like Bayesian dose escalation designs (from 48% in 2021 to 75% in 2024) [234].

In oncology, big data plays a role in creating trials based on genomic and biomarker designs that share a molecular alteration, especially to test drugs for pan-cancer activity [235,236]. However, translation of the available molecular data into prospective clinical trial design continues to lag behind the Volume of data being generated. Fragmentation among genomic, clinical, treatment and outcome datasets, variable data quality and annotation, and limited validation of many candidate biomarkers restricts the conversion into prospective enrollment criteria. Thus, the challenge is increasingly not identifying molecular differences between tumors but determining which differences reliably predict therapeutic response.

The quantitative case for biomarker-guided enrollment is direct--in the oncology success-rate analysis underpinning this section, biomarker-based patient selection in clinical trials from 2005–2015 raised the overall clinical trial probability of success from 1.6% to 10.7%, with the greatest effect occurring in phase III to approvals (33.6% to 63.6%) [161]. In a more recent publication covering 2011–2020, the overall probability of success jumped from 7.6% to 15.9% with biomarker preselection, with the greatest effect occurring in phase II trials (28.3% to 46.3%) [237]. These observations do not establish that the biomarker was the sole cause of higher success rates, but they demonstrate how molecular data can acquire clinical Value when they can reproducibly affect patient selection.

Given the large number of oncology drugs in clinical trials and the limited number of available patients for these trials, especially those presenting specific biomarkers, efficient trial designs including basket trials are incredibly important. NCI-MATCH is the largest effort of basket trials to date. Launched in 2015 and ended in 2023, it was a tumor-agnostic precision-oncology trial design with approximately 6000 patients screened in just 14 months. A rule-based decision tool (MATCHBox) served to automate the assignment of patients to 38 sub-studies and achieved a match rate of 18% with the use of a common NGS assay across the four labs [236,238]. Although most sub-studies closed without meeting the minimum signal threshold, 38% of the patients had an actionable alteration with one arm of the study returning a valuable result, such as dabrafenib with trametinib in BRAF V600E tumors generating a tumor-agnostic FDA approval [238]. This return is modest against the screening effort, and the limiting factor was not the assay but the limited number of available patients with the required alteration for the substudy [235]. Shared assays, definitions, and the decision tool can provide sufficient Vernacular to assign patients, but they cannot ensure success if insufficient biomarker cases exist.

Even when an appropriate trial is identified, enrolling the patient creates another bottleneck. Only 7.1% of adults with cancers participate in clinical trials, and consequently, poor enrollment accounts for 14.7% (phase I) and 35.5% (phase II and III) of early trial termination [239,240]. With eligibility criteria and patient histories living as free text rather than in a database with shared Vernacular, the Variety of descriptors causes a mismatch. A systematic review showed that using natural language processing and AI for trial enrollment studies (10 studies with more than 50,000 patients across 19 databases) led to comparable or superior results compared to manual review. In one study, adding natural language processing shrank post-screening review by 85% [241].

Despite the improvement in identification of patients for clinical trials, low enrollment persists due to eligibility nuances, availability of slots in clinical trials, clinician and patient preferences, geographic access, and administrative, infrastructure, and staffing burdens that limit trial availability at many treatment centers [242,243]. AI can improve patient identification but cannot overcome physical obstacles. A more profound obstacle is the lack of a consistent Vernacular within clinical trial documentation where EHR fragmentation degrades data availability (Section 6) [244].

### 5.3. External and Synthetic Control Arms

As discussed in the previous subsection, precision-oncology populations are small, so single-arm trials with surrogate endpoints, defined by the Response Evaluation Criteria in Solid Tumors (RECIST), have become common for accelerated approval despite forgoing the traditional comparison to a standard of care [245,246]. These trials can incorporate real-world and historical data as the missing connection based on previously conducted clinical trials, EHR data, and retrospective observational cohort studies. Erdafitinib used real-world longitudinal patient EHR data as a comparator to be granted accelerated approval for fibroblast growth factor receptor genetic alteration in unresectable urothelial cancer [245,247]. Similarly, the FDA approved blinatumomab for patients with precursor B-cell acute lymphoblastic leukemia using a single-arm BLAST trial, which detected minimal residual disease and drew its comparator from a retrospective observational cohort [245,248]. New variants use generative adversarial networks (GANs) to construct synthetic control arms to reproduce the statistical properties of real-world datasets reducing the need for direct sharing of patient level EHR data [249].

Regulators have formalized expectations for these designs. In 2023, the FDA issued a draft guidance on the design and conduct of externally controlled trials through CDER, CBER, and the Oncology Center of Excellence [250]. The European Medicines Agency announced development of a reflection paper on external controls in 2026 [251]. The ICH M15 FDA Model-Informed Drug Development (MIDD) draft guidelines for strategic modeling and simulations include agent-based systems and AI/ML for decision-making and evaluation. Regulatory acceptance depends on the intended use of the model and the quality of the underlying data, assumptions, and model evaluation [252]. These frameworks describe how to constrain bias rather than how to remove it.

Data fragmentation, missing clinical characteristics, and inconsistent annotation can limit the ability to match or balance the populations and can introduce residual bias [245]. Two of the four dimensions applied to screening data in Section 4.6 recur here. RECIST endpoints against treatment-driven imaging schedules break semantic alignment and case report forms with unstructured notes break representational alignment, so the trial dataset and its external comparator resist joint analysis. Causal-inference approaches, including target trial emulation, can further structure comparisons using observational RWD by defining eligibility criteria, treatment strategies, follow-up, outcomes, and analysis before the data are evaluated [36]. No primary analysis in FDA oncology applications has rested on formal external control comparison, so retrospective external controls have only reached regulatory validation in supporting roles, while GAN-generated synthetic controls remain a computational proof of concept [249]. As a result, a large Volume of RWD has limited Value if an appropriate comparator population cannot be constructed. These limitations keep external and synthetic controls supplemental rather than general substitutes.

### 5.4. Adaptive Monitoring of Disease and Clinical Trial Endpoints

Precision oncology populations are small and heterogeneous, so clinical trial designs that are adaptable based on the incoming data, rather than the traditional two-arm clinical trial designs, are increasingly used. The ICH E20, a draft guidance on Adaptive Designs for Clinical Trials, defines an adaptive clinical trial as one that permits planned modifications to sample size, treatment arms, enrolled subpopulations, and participant allocations based on ongoing analysis of accumulating trial data. Sponsors must pre-specify these modifications in the protocols and control error probabilities [253].

In oncology, this adaptive logic has developed over the past two decades with a single adaptive clinical trial able to transition from learning to confirmatory stages using the modifications listed above [254]. Two different types of adaptation classes are common: (a) group sequential designs can stop clinical trials early for efficacy or lack thereof and re-estimate the sample-size if an effect seems promising but has insufficient data [255], or (b) Bayesian response-adaptive randomization continually adapts assignment towards the better-performing arms using predictive probabilities. The algorithm learns from accumulating outcomes to inform the optimal treatment assignments [256]. This adaptive approach is used in Investigation of Serial Studies to Predict Your Therapeutic Response with Imaging and Molecular Analysis (I-SPY2) clinical trials such as the reclassified sequential multiple assignment randomization trials [257], which include biomarker-driven protocols like those discussed in Section 5.2. Adaptive clinical trials depend on a high Velocity of data, as outcomes must be measured and retrieved from the data sources quickly to inform the following decisions. The efficiency of an adaptive clinical trial erodes with missing data or without explicit control for temporal trends [258].

The Volume and Variety of data are shaping the endpoints of clinical trials. RECIST-based standardized responses (Section 5.3) classify complete or partial response to therapeutic, stable, or progressive disease derived from the diameters of target tumors [246]. As each of these criteria can also be affected by human inputs, AI has been used to automate the components of RECIST through tumor detection and measurements [259,260]. In a multicenter study, follow-up chest-abdomen-pelvis computed tomography (CT) were examined by both experts and trained AI. The AI assistance reduced per-patient read time and raised RECIST outcome agreement by 7.7 percentage points for a modest increase in Variability of lesion size. Expert reader assistance raised it by 13.3 percentage points, showing that automation supports supervised rather than fully autonomous reading [261]. Beyond RECIST, radiomics has been applied to other imaging criteria for shape, texture, and intensity features using routine diagnostic imaging techniques such as CT, magnetic resonance imaging (MRI), and positron emission tomography (PET) and has been proposed as an integrative imaging endpoint that captures response signal misses [262]. Veracity constrains this extension, as across multicenter rectal MRI, hardware and acquisition differences explained 64.3% of variation in the mean apparent diffusion coefficient compared to 0.4% by patient factors.

### 5.5. In Silico Clinical Trials and Digital Twins

In silico clinical trials and digital twins are emerging as complementary approaches to conventional clinical testing rather than replacements. In silico clinical trials are based on computational testing, using models informed by the large Volume of data previously generated in past trials, and digital twins are defined by the FDA as a virtual patient-specific replica based on the patient with inputs based on physical state and endowed with predictive capability to inform decisions [263,264]. The digital twin is a virtual twin of every individual for personalized care, population-level evidence, and, in this scenario, providing a cohort for novel therapeutic testing in an in silico virtual clinical trial [264].

In oncology, longitudinal clinical, molecular, imaging, and treatment response data could allow digital twins to be updated with disease progression. Proposed applications would include treatment selection, dose optimization, monitoring therapeutic response, and predicting treatment resistance [263,264,265]. In this way, digital twins could use information generated during treatment to inform subsequent clinical decisions rather than functioning only as static virtual participants in a clinical trial. Chen et al. identified 202 publications and 48 registered trials involving in silico clinical trial methods, but only 76 publications and 19 trials were directly related to drug developments. Neoplasms were among the most common application areas with 43 drug development publications. Overall, only 14 of the 48 registered trials were completed, showing how in silico trials are still a new area [266].

The Veracity of any such digital twin is based on the design. The two common strategies are physics-based mechanistic models of biology (computationally slow and makes inferences based on incomplete mechanistic knowledge) or data-driven AI models (work with incomplete information leading to poor extrapolation or poorly interpretable ML models) [265]. Mechanistic models based on partial-differential equations calibrated to imaging have forecast breast cancer progression and ordinary differential equation models of low-grade glioma response to temozolomide fitted to longitudinal imaging to create a virtual cohort for comparing dosing schedules [263,266]. Hybrid approaches combining mechanistic and data-driven modeling are increasingly being investigated to address the limitations of either methodology alone [265].

As with virtual screening in the preclinical pipelines (Section 4.4), model fidelity and validation constrain in silico clinical trials and digital twins, but they are nevertheless emerging as useful tools to triage and simplify clinical trials by improving the clinical trial design and reducing required enrollment sizes. The models behind digital twin models are not yet fully realized due to gaps in understanding disease in terms of the complex biology of a human patient. Neither increasing data volume nor computational sophistication provides a complete representation of a human patient. Incomplete biological knowledge, sparse longitudinal measurements, and limited prospective validation constrain the reliability of patient-specific predictions [265].

Nitschke et al. proposed that combining digital twin models with knowledge graphs improves performance [267]. However, improved integration does not remove the need for independent validation. For now, digital twins are best considered hypothesis generation tools rather than substitutes for clinical trials or true twins.

## 6. Cancer Drug Utilization: Data from Real-World Use

The information utilized in the drug discovery and clinical trial process traces back to the collection of real-world data (RWD) from healthcare records. RWD is the raw data relating to patient healthcare, while real-world evidence (RWE) is the clinical evidence from the analysis of RWD and can be used to inform the development of biological models, chemical classes, and the clinical testing for new therapeutics. Post-approval RWD can also identify adverse events patients experience in routine use, informing safety, dose refinement, and therapeutic utilization [268,269]. In this review, RWD has been mentioned in Section 5.3 as a comparator for single-arm oncology trials, but much of the use case of RWD is post-drug approval [268,269]. RWD and RWE close the loop between discovery, approval, and utilization, and data generated at one stage of the pipeline gains additional Value when researchers can integrate it into earlier stages.

### 6.1. Electronic Health Records

Healthcare records and billing databases are a major source of RWD. These databases have a large volume of data for use in oncologic drug discovery; however, they are limited due to questions about Veracity, Variability, and a lack of shared Vernacular. Analysis of EHRs to obtain RWD and RWE has been used for optimization of dosing regimens, cost evaluation in specific populations, defining clinical trial endpoints, establishing biomarkers of interest, and discovering potential secondary uses of drugs [270]. The burden of data entry into EHRs primarily falls on physicians, clinical staff, or, as of late, LLMs [271,272,273]. Errors, missing information, and inconsistently recorded information can therefore propagate into subsequent RWD analyses. The loss is measurable and falls on the data most needed. Tracing lung cancer patients from the source EHR to an institutional registry and into FHIR extracts found concordant demographics across all three. The biomarker data present in the EHR was missing from 80–100% of FHIR extracts [274].

One approach to reducing some of the limitations of RWD and RWE from EHRs is the consolidation under a single umbrella and methodology for inputting data. The US Department of Veteran’s Affairs (VA) migrated to a single EHR platform, linking both active military EHR with VA EHR due to the EHR Modernization Effort [275]. This provides clinicians more complete data and has led to the Veterans Precision Oncology Data Commons (VPODC) listed in Table 3, which integrates deidentified clinical, genomic, and imaging data for oncology research [84].

Beyond the VA, EHR development in the US has been primarily driven via federal incentives and is delivered by private industry where records are organized around insurance billing [276]. The competing systems have different data standards and Vernacular, and interoperability is poor, leading to inefficient use cases for RWD [277]. Across the 53 member states of the World Health Organization (WHO) European region, implementation of an EHR started in 1992 and (mostly) finished by 2022, but issues of interoperability and a lack of consistent data standards remain [278]. The lack of interoperability and consistent data standards have resulted in relatively few research-ready EHRs, with one of the exceptions being the United Kingdom (UK), where the Clinical Practice Research Datalink has linked anonymized primary records for research use [279]. Worldwide healthcare records therefore remain a patchwork, and the large Volume of healthcare data does not automatically translate into Value when differences in structure, terminology, and data quality prevent reliable integration.

### 6.2. Real-World Evidence Based on Real-World Data

The 21st Century Cures Act of 2016 directed the FDA to evaluate the use of both RWD and RWE, leading to a FDA framework and guidance on the use of RWD and RWE to support regulatory decisions for drugs and biological products [280]. As of June 2026, there are 13 drugs and 9 biological products that used RWE for approval [281,282]. RWE does not replace randomized controlled clinical trials but rather supplements them, especially in underpowered trials. For example, on 4 April 2019, the FDA expanded the label of palbociclib (Ibrance) for Hormone Receptor-positive, HER2-negative advanced metastatic breast cancer in combination with an aromatase inhibitor in men [283]. Despite breast cancer being the most prevalent malignancy in women (US lifetime frequency of 13%), it is extremely rare in men (lifetime frequency of 0.1%) [284]. The rarity of breast cancer in males precludes the conduct of large randomized clinical trials, and historically, as indicated by the FDA, males were excluded from breast cancer drug trials. For approval for use in men, FDA’s favorable benefit/risk assessment relied predominantly on the data collected in women, the palbociclib global safety database consisting of 362 cases of male breast cancer with 752 reported adverse events, and a retrospective analysis of 12 palbociclib-treated males with complete radiological reports within EHRs [283]. This provides a direct example of RWD supplementing existing clinical trial evidence to support a regulatory label expansion in a population that was difficult to study prospectively.

As RWD are observational, associations between treatments and outcomes do not necessarily represent causal treatment effects. Differences in disease severity, treatment selection, comorbidities, and other patient characteristics can confound comparisons. Two failure modes recur in oncology. First, confounding by indication is where the reason a treatment was chosen also predicts the outcome. Second, immortal time bias is where the interval before treatment starts cannot contain the outcome of interest during a follow-up time [36]. Causal-inference approaches, including target trial emulation, attempt to reduce these biases by defining eligibility criteria, treatment strategies, follow-up, outcomes, and analysis in a manner analogous to a hypothetical randomized trial [36]. These methods can strengthen RWE, but they remain dependent on adequate measurement of confounders and sufficiently consistent data across sources.

RWD can support novel indications for existing drugs through repurposing. Randomized controlled studies of cardiovascular prevention linked aspirin, which predates the FDA, to cancer outcomes [285]. The pooled analysis demonstrated a decrease in the risk of colorectal cancer by 24% and the associated mortality by 35% after 8–10 years [285]. This has been confirmed prospectively with a randomized Adjuvant Low Dose Aspirin in Colorectal Cancer (ALASCCA) trial that showed the impact of low-dose aspirin on localized colorectal cancer caused by PI3K pathway alterations [286]. This progression from an observed association to biomarker-selected prospective testing demonstrates the utility of information gathered later in a drug’s lifecycle and its feedback into clinical development. RWE and RWD are providing new solutions and information to the oncology drug development industry, especially in designing clinical trials and providing insights of secondary uses for drugs.

### 6.3. The Role of Electronic Health Records in the Analysis of Real-World Data to Form Real-World Evidence

One of the biggest challenges to the utilization of RWE is the lack of harmonization in EHRs, a problem described in Section 3.1. Beyond the Variability, the lack of shared Vernacular between EHRs makes it difficult to auto-populate select EHR data into research databases, especially once the protected health information (PHI) is de-identified [287,288].

Overcoming this depends as much on people as it does on infrastructure. To aid in this research, in 2015, the VA and the NCI launched the Big Data-Scientist Training Enhancement Program (BD-STEP) to produce clinically-focused data scientists who would be able to improve healthcare through innovative data science methods [289], a need referenced and discussed further in Section 7. Scientists trained in big data allow for large healthcare systems to better integrate clinical and molecular information. The Applied Proteogenomic OrganizationaL Learning and Outcomes (APOLLO) network, for examples curates and translates EHR data to RWD and RWE [290].

The FDA Oncology Center of Excellence has used similar large datasets for earlier submission of priority results and datasets to compress the review timeline itself. The Real-Time Oncology Review (RTOR) pilot, initiated in 2018, supports supplemental drug applications to add new indications, dosing regimens, NDA, and biological license applications. A pilot running from 2018–2020 that supported 20 oncology applications demonstrated a median approval time of 3.3 months [291]. All 20 received priority review, but the FDA authors note that the shorter timeline may reflect the more straightforward nature of the applications rather than the review process itself [291]. Through 2023, RTOR has since supported 76 of 363 new oncology indication approvals [292]. RTOR therefore aids the clinical trial efficiencies mentioned in Section 5 from the review side. Large RWE frameworks, such as APOLLO, and the increasing Volume and Velocity of regulatory information provided to the FDA, show the importance of education for more and better data science.

## 7. Big Data, Artificial Intelligence, and Education

One of the constraints in the utilization of big data and AI has been a limited work force trained in oncology, drug discovery, and data science. Most data scientists have experience with large datasets but lack the biological and experimental knowledge to fully utilize the information. The experimental and clinical scientists have the biological background but lack the data science knowledge. A workforce split along these skillsets lacks a shared Vernacular and thus makes collaborative, cross-functional teams inefficient and costly. In this section, we outline the skillsets necessary to work with big data and AI in oncological drug discovery, with representative self-directed resources in Table 9 to enable efficient use of existing datasets, models, and analysis of generated data.

### 7.1. Foundational Data Science Skills

Programming, applied statistics, and ML form the foundation of data science for analyzing big data [293]. The most common data science languages are Python and R, with multitudes of community-driven packages to support science, such as those in Section 3. LLMs such as ChatGPT, Claude, and others have lowered the barrier of entry, allowing bench scientists to create more complex programming-based data pipelines without years of experience [83]. However, this does come with the caveat of potentially decreased Veracity and Value of the analyzed data due to a lack of code and pipeline validation. A benchmark of 293 coding tasks from 39 biomedical studies spanning biomarkers, genomic profiling, therapeutic response and pan-cancer analysis found overall accuracy below 40% on eight proprietary and eight open-source models [294]. Thus, foundational data science skills must also emphasize reproducibility, version control, and documentation in addition to analysis.

### 7.2. Skills for Initial Discovery

Preclinical discovery is a blend of large datasets, both cheminformatics and bioinformatics, and structural computation. The practical skills build upon the foundational skills (Section 7.1) to navigate and effectively utilize the data commons and visualization tools from Table 3 and Table 4. Structure-based workloads require the retrieval and analysis of predicted models to run docking and molecular dynamics simulations. To use these tools appropriately, the researcher must understand both the biological question and underlying tool assumptions, emphasizing the importance of fluency in both data science and biological Vernacular. Both cheminformatics and bioinformatics have a culture of large, open-source datasets and teaching materials maintained by both institutions and individuals through annotated notebooks and code-rich tutorials. The same open-source tooling culture extends to biologics, where structure prediction and de novo design toolkits have made antibody and protein engineering approachable like small-molecule drug discovery. The Open Molecular Software Foundation helps researchers bring these open-source tools to their discovery efforts [295].

### 7.3. Skills for Clinical Trials and Real-World Data

The same foundational skills (Section 7.1) carry over for use in clinical trials and RWD. The result is a blend of biostatistics, causal inference, and health data informatics while also controlling for Variability. Biostatistics and adaptive clinical trial design (Section 5.4) form the core, supplemented by observational data for target clinical trial emulation (Section 6.2), with EHRs adding informatics complexity. Researchers must be familiar with both clinical questions and the biases in the medical records, emphasizing fluency in clinical, regulatory, and data science Vernacular. Those biases are specific and learnable, with biomarker fields disappearing almost entirely downstream (Section 6.1), so a trainee not familiar with extracted dataset bias can form incorrect conclusions not supported by underlying data.

**Table 9:** An example list of computational resources for learning more about handling big-data and AI/ML drug discovery resources.

Category	Resource	Provider/Source	Focus
Foundations	MIT OpenCourseWare [296]	Massachusetts Institute of Technology (MIT)	Free MIT course materials in math, statistics, computer science, computational biology, etc.
	DeepLearning.AI [297]	DeepLearning.AI	Structured machine learning and deep learning
	Kaggle Learn [298]	Kaggle	Competition-based practice on data science challenges on public datasets
Target Identification	European Bioinformatics Institute (EMBL-EBI) Training [299]	EMBL-EBI	Tutorials, webinars, and tutorials for using ChEMBL, Ensembl, Protein Database (PDB), data storage, and other resources
	Rosalind [300]	Rosalind.info	Learning resource for genomics and sequence analysis via example problem sets
	cBioPortal [97,98]	Memorial Sloan Kettering Cancer Center, cBioPortal Consortium)	Exploration and visualization of cancer genomics for target discovery
Cheminformatics and molecular ML	RDKit [97]	Open source Community	A foundational open-source cheminformatics toolkit with example workflows
	Practical Cheminformatics tutorials and blog [301]	Community lead by Patrick Walters	Tutorials and blogs on cheminformatics and ML with example notebooks and code
	TeachOpenCADD [302]	Volkamer Group at Universitätsmedizin Berlin	Lots of tutorials around computer-aided drug design, similarity docking, ML, pharmacophores
	DeepChem [303]	DeepChem Community	Python library and tutorial for ML in chemistry: property prediction and generative design
	ATOM Modeling Pipeline (AMPL) [304]	ATOM Consortium	Tutorials series on how to use the ML pipeline. Extends DeepChem
Biologics and protein design	PyRosetta [305]	RosettaCommons	Python interface and learning notebooks for protein modeling and de novo design
	Hugging Face Learn [306]	Hugging Face	Free courses on transformers, on Evolutionary Scale Modeling family models and protein language modeling
	AlphaFold: A practical guide [307]	EMBL-EBI	Free tutorials designed with Google DeepMind on how to use AlphaFold in research. Many more tutorials on AlphaFold2/3 also exist
Clinical trials and real-world data	Causal Inference: What If (Book) [36]	Harvard University	Free textbook with causal inference and target trial emulation data
	Project Data Sphere [308]	Project Data Sphere	Open de-identified historical oncology trial data for control-arm and RWD practice

## 8. Conclusions

This review has organized the impact of big data and AI on cancer drug discovery using a framework of eight Vs: Volume, Velocity, Variety, Veracity, Value, Variability, Visualization, and, as we proposed in this review, Vernacular, through the entire oncology drug discovery pipeline [22,23,24,309]. The review addressed three interrelated aims. First, the framework demonstrates that the preclinical, clinical, regulatory, and post-approval stages are connected through the data they consume and generate rather than independent processes. Second, we proposed Vernacular as the eighth “V” to describe why datasets that may be individually curated can be difficult to interpret and analyze together. Third, the examples examined throughout the pipeline support that data integration, rather than data generation alone, is increasingly a binding constraint in drug discovery. The growing Volume, Velocity, and Variety of available data provide opportunities at each discrete step, but their downstream Value depends on Veracity and on whether information can be reliably interpreted and integrated.

AI increasingly supports this connected pipeline by prioritizing rather than replacing experimental and clinical work. Computational models can rank targets, compounds, structures, synthetic routes, biomarkers, patients, and trial strategies, including dose optimization and adaptive designs, before researchers commit resources to validation. Experimental and clinical results then generate new data that refine later models. Real-world use extends this feedback loop by revealing safety signals, dose-response patterns, resistance, and potential new indications [193,203,206,207,208]. Cancer drug discovery therefore functions less as a linear sequence and more as an iterative design-build-test-learn system.

Rapid advances in ML, multimodal models, foundation models, and LLMs do not guarantee proportional gains in drug discovery. Biological and pharmacological data depend strongly on experimental system, assay design, disease state, and patient population. Adding more heterogeneous observations can introduce noise, bias, and confounding instead of improving prediction. Recent critical assessments similarly argue that AI has produced impressive benchmark results but limited evidence of improved drug-development decisions [227]. The field should therefore ask not only whether a model predicts well but also whether it generalizes to its intended use and improves a meaningful downstream decision.

This is especially important as private organizations train leading models on proprietary datasets with limited transparency to judge model effectiveness. Retrospective benchmark performance is necessary but not sufficient when a model influences experimental, clinical, or regulatory decisions; external validation, prospective evaluation, calibration, reproducibility, and characterization of failure modes deserve equal weight as measures of Value and Veracity. Model access complicates this. The Chai-1 authors could not benchmark against AlphaFold3 because its license forbids commercial evaluation [310] and the Co-Scientist authors did not release source code, citing proprietary infrastructure, computational cost, and safety [311]. Open weights, transparent evaluation sets, and shared benchmarks therefore remain necessary counterparts to proprietary development.

Public and private data are also continuing to converge. The NIH and EBI data commons remain the most common public resources for multi-omic and chemical data, but public-private partnerships such as ATOM and federated approaches like MELLODDY (Section 3.3) are increasingly bringing previously proprietary data into shared modeling [73,74]. However, access alone does not resolve differences in assay design, terminology, molecular representation, annotation, provenance, or patient populations. Federated learning, for example, can enable joint model training without centralizing proprietary data, but it cannot make heterogeneous datasets semantically equivalent.

Regulatory expectations for AI-generated evidence increasingly center on whether a model is credible for its intended decision. FDA draft guidance applies a risk-based framework requiring a defined context of use and evidence sufficient to establish model credibility [312]. EMA’s reflection paper on AI across the drug product lifecycle emphasizes risk-based performance assessment, transparency, predefined performance criteria, monitoring for model drift, and re-evaluation after model changes [313]. In January 2026, the FDA and EMA put out a joint guiding principles of good AI practice in drug development aligning on data governance, performance assessment, and lifecycle management [314]. Regulatory acceptance therefore depends on demonstrating model fitness for a specific decision and maintaining that credibility across the model lifecycle.

Regulatory agencies are also catching up and putting out updated guidelines and tools for the effective use of big data to improve drug discovery. The FDA has deployed internal AI by pairing the agency-wide Elsa, an AI currently used to accelerate scientific review, safety, signal triage, and label comparison, with the Harmonized AI and Lifecycle Operations for Data (HALO), combining over 40 disparate application and data submission systems to ease data handling and the creation of workflows [315,316]. The FDA is also providing new draft guidance documents for quantitative systems pharmacology (QSP)-based dose selection for the minimum anticipated biological effect level (MABEL) for first-in-human clinical trials [317]. MABEL formalizes the use of model-informed dosing, potentially extending in silico use instead of purely animal models. This builds on the revised draft guidance, demonstrating substantial evidence of effectiveness for human drug and biological products, to potentially allow real-world and mechanistic data to become an accepted basis for approval [318].

These findings clarify why we propose Vernacular. Vernacular does not replace FAIR principles, controlled vocabularies, ontologies, OMOP, CDISC, FHIR, or related standards. Instead, it describes a property of the data ecosystem: the extent to which independently generated datasets align in semantics and terminology, representation, exchange and governance to support joint analysis without repeated source-specific curation. Existing standards and frameworks can therefore increase shared Vernacular rather than compete with it.

The promise of big data and AI in cancer drug discovery will therefore depend less on scale itself than on whether the field can integrate, interpret, and trust the information at that scale.

## Figures and Tables

**Table 1:** The eight-V framework used throughout this review.

Property Class	Term	Definition	Application in Cancer Drug Discovery
Descriptive	Volume	The amount of data	Large databases containing multi-omic, imaging, screening, electronic health records, etc.
	Velocity	The rate of data generation	Generation of data in silico, in vitro, and in vivo
	Variety	The diversity in types of data	Genomic, imaging, chemical, clinical, etc. data types
Constraining	Veracity	The accuracy and trustworthiness of the data	Assay noise, false positives, clinical recording errors, bias.
	Variability	The differences in data over time and sources	Tumor heterogeneity, batch effects, assay differences, and changing clinical populations
	Visualization	The representation of data in interpretable form	Graphical representation to make patterns interpretable
	Vernacular (proposed)	The degree of semantic, representational, exchange, and governance alignment	Determines whether independently generated datasets can be jointly utilized across the oncological drug discovery pipeline
Outcome	Value	The usefulness of the data for the intended use given the constraining properties	Whether the available data is sufficient to nominate a target, qualify a biomarker, select a dose, or support approval

**Table 2:** Major artificial intelligence approaches applied across the oncological drug discovery pipeline and the typical validation reached by examples addressed in this review.

Approach	Oncology Use	Advantage	Limitation	Typical Validation
Supervised	Quantitative structure–activity relationships (QSAR) ligand prediction (Section 4.3) ADMET prediction (Section 4.4) Trial outcome prediction (Section 5.1)	Quantitative prediction	Label bias and leakage Weak sensitivity under independent evaluation (Section 5.1)	Computational to Experimental
Unsupervised	Chemical–space clustering (Section 4.2) Subtype discovery and patient stratification (Section 5.2)	Finds latent structures without labels	Biological meanings can be uncertain (Section 2.4)	Computational to Experimental
Reinforcement	Retrosynthetic route search; De novo design (GENTRL) (Section 4.5)	Optimization towards a defined object	Reward misspecification (Section 4.5)	Computational to Experimental
Generative	Molecule design (Section 4.5) Antibody design (Section 4.7)	Explores new chemical space	Validity/ Synthesizability (Section 4.5)	Computational to Experimental
Foundation/ multimodal	Integrated chemical, omic, imaging, and clinical tasks (Section 2.2, Section 4.6 and Section 5.1)	Transfers across tasks. Can work on low label tasks	Provenance, heterogeneity	Computational. Prospective. Varies depending on task

## Data Availability

No new data were created or analyzed in this study. Data sharing is not applicable to this article.

## Abbreviations

The following abbreviations are used in this manuscript:

ADMET	absorption, distribution, metabolism, excretion, and toxicity
AI	artificial intelligence
ATOM	Accelerating Therapeutics for Opportunities in Medicine
CBVLS	compound-based virtual ligand screening
CDISC	Clinical Data Interchange Standards Consortium
CRDC	Cancer Research Data Commons
CTD	Common Technical Document
DL	deep learning
EBI	European Bioinformatics Institute
EHR	electronic health records
EMA	European Medicines Agency
EMBL	European Molecular Biology Laboratory
EU	European Union
FAIR	Findable, Accessible, Interoperable, and Reusable
FDA	Food and Drug Administration
FHIR	Fast Healthcare Interoperability Resources
GDC	Genomic Data Commons
GDPR	General Data Protection Regulation
HCS	high content screening
HIPAA	Health Insurance Portability and Accountability Act
HTS	High-Throughput Screening
ICH	International Council of Harmonisation
InChI	International Chemical Identifier
LLM	large language model
MCTS	Monte Carlo tree search
MIDD	Model-Informed Drug Development
MLPCN	Molecular Library Probe Production Centers Network
ML	machine learning
NCI	National Cancer Institute
NDA	New Drug Application
NIH	National Institutes of Health
OMOP	Observational Medical Outcomes Partnership
PDB	Protein Data Bank
PHI	protected health information
QSAR	quantitative structure–activity relationships
RBVLS	receptor-based virtual ligand screening
RECIST	Response Evaluation Criteria in Solid Tumors
RTOR	Real-Time Oncology Review
RWD	real-world data
RWE	real-world evidence
SMILES	Simplified Molecular Input Line Entry System
TCGA	The Cancer Genome Atlas
US	United States
USD	United States dollar
VA	U.S. Department of Veterans Affairs
VLS	virtual ligand screening
